# Interaction between microbiota and immunity in health and disease

**DOI:** 10.1038/s41422-020-0332-7

**Published:** 2020-05-20

**Authors:** Danping Zheng, Timur Liwinski, Eran Elinav

**Affiliations:** 1 Immunology Department, Weizmann Institute of Science, 234 Herzl Street, 7610001 Rehovot, Israel; 2 Department of Gastroenterology, The First Affiliated Hospital, Sun Yat-sen University, Guangzhou, Guangdong China; 3 1st Department of Medicine, University Medical Center Hamburg-Eppendorf, Hamburg, Germany; 4 Cancer-Microbiome Division, Deutsches Krebsforschungszentrum (DKFZ), Neuenheimer Feld 280, 69120 Heidelberg, Germany

**Keywords:** Immunology, Molecular biology

## Abstract

The interplay between the commensal microbiota and the mammalian immune system development and function includes multifold interactions in homeostasis and disease. The microbiome plays critical roles in the training and development of major components of the host’s innate and adaptive immune system, while the immune system orchestrates the maintenance of key features of host-microbe symbiosis. In a genetically susceptible host, imbalances in microbiota-immunity interactions under defined environmental contexts are believed to contribute to the pathogenesis of a multitude of immune-mediated disorders. Here, we review features of microbiome-immunity crosstalk and their roles in health and disease, while providing examples of molecular mechanisms orchestrating these interactions in the intestine and extra-intestinal organs. We highlight aspects of the current knowledge, challenges and limitations in achieving causal understanding of host immune-microbiome interactions, as well as their impact on immune-mediated diseases, and discuss how these insights may translate towards future development of microbiome-targeted therapeutic interventions.

## Introduction

The human body, including the gut, skin and other mucosal environments, is colonized by a tremendous number of microorganisms, collectively termed the microbiome.^1^ The collective genomes of bacteria and other microorganisms in this ecosystem, including fungi, viruses, parasites,^2^ have been increasingly investigated during the past two decades, facilitated by a rapid development of culture-independent genomic techniques. Recent advances in microbiome research revealed that the gut microbiome is not just a passive bystander, but actively impacts multiple host functions, including circadian rhythmicity, nutritional responses, metabolism and immunity.^3,4^

The mammalian immune system encompasses a complex network of innate and adaptive components in all tissues, and plays a vital role in host defense against various potentially harmful external agents and endogenous perturbations of homeostasis. From an ecological perspective, mammals and their commensal microorganisms co-evolved toward mutualism and hemostasis.^5^ Such intimate relationship requires the proper functioning of host immunity to prevent commensals from over-exploitation of host resources while maintaining immune tolerance to innocuous stimuli.^6,7^ However, perturbation of the gut microbiome by environmental incursions (such as antibiotic use, diet or changes in geography), impairment of host-microbiome interfaces, or alterations of the immune system can result in systemic dissemination of commensal microorganism, susceptibility to pathogenic invasion, and aberrant immune responses. In addition to regulation of infection and commensal spread, microbiome-immune interactions are implicated in a variety of ‘non-communicable’ gastrointestinal diseases including inflammatory bowel disease (IBD)^8^ and celiac diseases,^9^ as well as extra-intestinal disorders ranging from rheumatic arthritis,^10^ metabolic syndrome,^11^ neurodegenerative disorder^12^ to malignancy.^13^ The interactions between the gut microbiota and host immunity are complex, dynamic and context-dependent. Here, we review and exemplify important current knowledge and key concepts linking the microbiome to development and function of the immune system. We highlight some of the existing mechanistic dissections of multifaceted microbiome-immunity dialogs in both homeostatic and diseased states. Moreover, we discuss the challenges and perspectives of microbiome-targeted strategies in studying disease pathogenesis and developing  new microbiome-related treatments. As the large body of evidence related to host immune-microbiome interactions cannot be summarized by a single review, we aim to provide key concepts and examples of such interactions and their potential effects on human health and disease risk, while referring throughout the review to multiple other recent reviews^14–16^ focusing on distinct aspects of these emerging interactions.

## The role of the microbiome in immune system development

Early-life colonization of the mammalian host’s mucosal surfaces plays a pivotal role in maturation of the host’s immune system.^17^ Most critical events in education of host immunity may take place during the first years of life, in which microbiota composition displays the highest intra- and inter-individual variability before reaching a more stable adult-like configuration at the age of ~3 years.^18–20^ However, the 'window of opportunity' thus created, may also render infants more susceptible to environmental incursions to the microbiota, with potentially long-lasting harmful impacts on immunity.^21^ The immaturity of the immune system in newborns and infants is highlighted by an increased susceptibility to various infectious pathogens,^22^ rendering infectious diseases the leading cause for mortality in children.^23^ On the other hand, an increased propensity towards excessive inflammation is also frequently encountered in prematurely born infants, as exemplified by the potentially devastating disorder necrotizing enterocolitis.^24^ Most studies to date have not noted a reproducible microbial colonization already occurring in utero,^25^ and it is generally believed that the largest share of colonization occurs after birth, mainly originating from the maternal microbiota.^26^ Multiple modulators impact this initial colonization, including delivery mode that impacts on the composition of the initial microbiota across multiple body habitats.^27^ It is well established that in neonates maternal antibodies delivered via breastmilk offer crucial passive protection against pathogens.^28^ Interestingly, a recent work showed that the commensal microbiota of pregnant mice drives antibody-mediated protective immunity through breastfeeding.^29^

The study of mechanistic causal relationships between commensal microbiota and host immunity is strongly informed by the use of germ-free (GF) animal models. Early studies on GF animals demonstrated that absence of commensal microbes is associated with profound intestinal defects of lymphoid tissue architecture and immune functions.^30^ αβ and γδ intra-epithelial lymphocytes (IELs) are significantly reduced in GF mice compared to conventional colonized animals, and can be strongly induced upon de novo colonization.^31^ IgA antibodies are a mainstay of protective humoral mucosal immunity and show substantial reduction in newborns and GF animals, which is rapidly restored by microbial colonization.^32^ Gestational maternal colonization increases intestinal group 3 innate lymphoid cells (ILC3s) and F4/80^+^CD11c^+^ mononuclear cells in the offspring.^26^ The lamina propria of the small intestine contains a large number of IL-17^+^CD4^+^ T (Th17) cells, which represent a class of potent immunomodulatory effector cells.^33^ Th17 cells are absent in GF mice and are inducible upon microbial colonization, most notably with segmented filamentous bacteria (SFB),^33,34^ but also other commensal bacteria.^35^ Induction of Th17 cells by SFB is enabled by their adhesion to epithelial cells.^36^ A bacterial polysaccharide derived from the ubiquitous commensal *Bacteroides fragilis* directs the maturation of the developing immune system in mice, including correction of systemic T cell deficiencies and Th1/Th2 imbalances in lymphoid tissues.^37^ An early B cell lineage in the intestinal mucosa is regulated by extracellular signals from commensal microbes that influence gut immunoglobulin repertoires.^38^ Intestinal microbial diversity during early-life colonization is critical to establish an immunoregulatory network that protects from induction of mucosal IgE, which is linked to allergy susceptibility.^39^ The innate immune receptor Toll-like receptor 5 (TLR5) serves as a sensor for bacterial flagellin. Although in mice TLR5-mediated counter-selection of colonizing flagellated bacteria is constrained to the neonatal period, this critical process shapes gut microbiota composition and thus impacts on immune homeostasis and health in adult life.^40^

To summarize, it is increasingly recognized that critical host immune-microbiota interactions operate during a critical time window in early life, which may have long-lasting impacts on multiple immune arms contributing to immune homeostasis and susceptibility to infectious and inflammatory diseases later in life. However, the mechanisms of these interactions are still relatively poorly defined, and the long-term impacts of subtler dysbiosis states during the neonatal period on adult immunity and risk of immune-mediated diseases merit future studies in human. More detailed insights into such modulatory effects, if present, may bear impact on understanding, prevention and treatment of immune-related disorders.

## Interaction between microbiota and immune system in homeostasis

### Host-induced compartmentalization of intestinal microbiota

The best-studied interface for host-microbiota interactions is the intestinal mucosa. A remarkable feature of the intestinal immune system is its ability to establish immune tolerance towards an enormous and constantly changing wealth of harmless microorganisms while concomitantly preserving immune responses against pathogenic infection or commensal intrusion into the sterile body milieu.^41^ In a healthy state, the host’s immune response to the intestinal microbiota is strictly compartmentalized to the mucosal surface.^42^ A single layer of epithelium separates the intestinal lumen from underlying tissues. Many mechanisms are employed to achieve microbiota compartmentalization. A dense mucus layer separates the intestinal epithelium from resident microbes.^43^ The mucus barrier is organized around the hyperglycosylated mucin MUC2. However, MUC2 not only offers protection by static shielding, but also constrains the immunogenicity of intestinal antigens by imprinting enteric dendritic cells (DCs) towards an anti-inflammatory state.^44^ Tight junctions are a critical structure in restricting trans-epithelial permeability. Microbial signals, e.g., via the metabolite indole, promote fortification of the epithelial barrier through upregulation of tight junctions and associated cytoskeletal proteins.^45^ In addition, secretory IgA antibodies and antimicrobial peptides (AMPs) maintain the mucosal barrier function (see below).^32,46^ Intestinal DCs are believed to play a critical role in compartmentalizing  enteric microbiota, through mechanisms involving sampling of gut bacteria for antigen presentation.^47^

### Crosstalk between the innate immune system and the microbiota

**Fig. 1:**
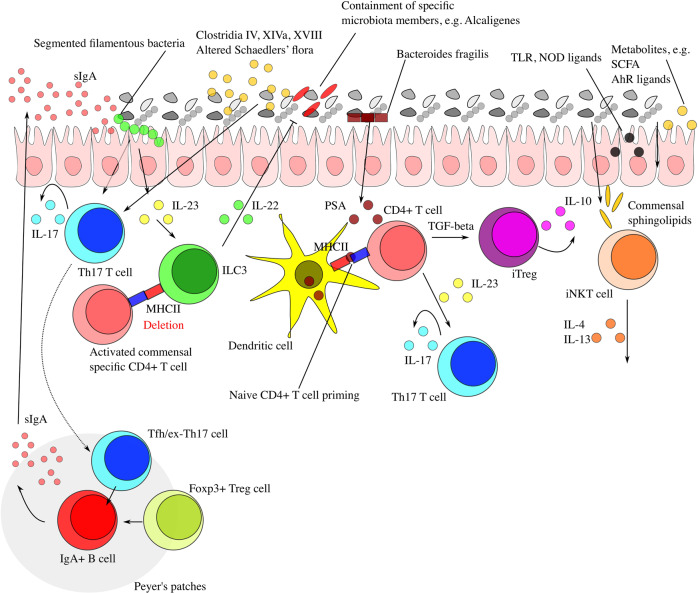
Intestinal microbiota-immunity interplay in homeostasis. Selected mechanistically well-characterized microbiota-immune system interactions are depicted. Microbiome-derived TLR and NOD ligands and metabolites (e.g., SCFA, AhR ligands) act directly on enterocytes and intestinal immune cells, but can also reach remote tissues via the systemic circulation to modulate immunity. Foxp3^+^ Treg cells and Tfh/ex-Th17 cells localize in Peyer’s patches to promote class switch of B cells and production of secretory (s)IgA. These contribute to compartmentalization of commensal microbiota and regulate homeostatic microbiota composition. Intestinal colonization by SFB and many other commensals promotes differentiation of CD4^+^ Th17 cells. Moreover, SFB colonization elicits signaling via the ILC3/IL-22/SAA1/2 axis to induce IL-17A production by RORγt^+^ Th17 cells. ILC3-derived IL-22 contributes to containment of specific microbiota members by promoting IL-17A production by Th17 cells. Furthermore, deletion of ILC3-expressed MHCII activates commensal-specific CD4^+^ T cells to prevent an immune response against harmless colonizers. Early-life microbial colonization limits the expansion of iNKT cells, in part via production of sphingolipids, to prevent potential disease-promoting activity within the intestinal lamina propria and the lungs. Colonization with *Bacteroides fragilis*, a prominent member of mammalian intestinal microbiota, is able to promote CD4^+^ T cell differentiation and to balance Th1 and Th2 populations, an effect that relies on its PSA. PSA is taken up by lamina propria DCs through a TLR2-dependent mechanism and presented to naïve CD4^+^ T cells. In the simultaneous presence of activated TGF-β, these cells can differentiate to regulatory T cells (iTreg). IL-10 produced by these cells promotes immune homeostasis. Contrarily, IL-23 licensed through the same cascade promotes expansion of pro-inflammatory Th17 cells.

Microbiota and innate immunity engage in an extensive bidirectional communication (Fig. 1). One of the phylogenetically oldest systems of innate immunity is represented by AMPs. The majority of intestinal AMPs is produced by Paneth cells, which represent specialized secretory cells of the small intestinal mucosa.^48^ Intestinal AMPs exhibit manifold interactions with the microbiota and are an essential component in shaping its configuration.^49^ Adding to the complexity of intestinal AMPs, antimicrobial secretion from pancreatic acini seems to be critical for maintenance of intestinal homeostasis, as mice featuring reduced secretion of pancreas-derived cathelicidin-related AMP secondary to lack of the potassium channel Orai1 demonstrate a dramatically increased mortality due to increased systemic microbial translocation and inflammation.^50^

Pattern recognition receptors (PRRs), such as Toll-like receptors (TLRs), were initially described to sense microbial signals during infection to elicit a protective immune response. However, ligands for PRRs are not exclusive to pathogens and are abundantly produced by commensal microbiota during healthy colonization (reviewed in^7^). TLRs are involved in host defense against pathogens, regulate the abundance of commensal microbes and maintain tissue integrity.^51^ TLR expression in the intestinal epithelium is characterized by a high diversity in terms of spatial, cell type-specific, and temporal patterns.^52^ TLR5 is of particular importance in shaping the gut microbiota,^53–56^ which might be confined to a critical time window during neonatal life.^40^ Polysaccharide A (PSA) produced by the commensal *Bacteroides fragilis* is another well-studied example of a single molecule promoting symbiosis and host immune system education.^57–59^ PSA is recognized by the TLR2/TLR1 heterodimer in cooperation with Dectin-1^60^, a C-type lectin PRR.^61^ Downstream to TLR1/TLR2 and Dectin-1 signaling, the phosphoinositide 3-kinase (PI3K) pathway is activated leading to inactivation of glycogen synthase kinase 3β (GSK3β), which in turn induces cAMP response element-binding protein (CREB)-dependent expression of anti-inflammatory genes.^60^ Moreover, Dectin-1 may regulate intestinal immunity by controlling Treg cell differentiation through modification of microbiota configuration.^62^ Additional PRRs suggested to shape the gut microbiota composition are NOD-like receptors (NLRs). Nucleotide-binding oligomerization domain-containing protein 1 (NOD1) serves as an innate sensor assisting generation of adaptive lymphoid tissues and maintenance of intestinal homeostasis.^63^ The bacterial sensor NOD2 prevents inflammation of the small intestine by restricting the growth of the commensal *Bacteroides vulgatus*.^64^ Stimulation of NOD2 by commensal bacteria promotes gut epithelial stem cell survival and epithelial regeneration.^65^

MyD88 is an adapter for multiple innate immune receptors that recognize microbial signals, and of the signaling pathways induced by the effector molecules interleukin-1 (IL-1) and IL-18 through their respective receptors.^66^ Mice deficient in MyD88 display an altered microbiota composition.^56^ MyD88 controls the epithelial expression of several AMPs, including RegIIIγ, which restricts the number of surface-associated gram-positive bacteria and limits activation of adaptive immunity.^67^ Moreover, MyD88 regulates T cell differentiation, promotes microbiota homeostasis through stimulation of IgA and controls the expansion of Th17 cells by restricting growth of SFB in mice.^68^

Some NLRs assemble into multiprotein complexes abundant in many different cell types termed inflammasomes, whose pleiotrophic immune functions are reviewed extensively elsewhere.^69^ Inflammasomes activate inflammatory caspases, which promote the maturation of IL-1β and IL-18, and induce a lytic type of cell death termed pyroptosis.^69^ NOD-, LRR (leucine‐rich repeat)- and pyrin domain-containing 6 (NLRP6) is such protein assembling inflammasome in the intestinal mucosa. The NLRP6 inflammasome has been linked with regulation of microbiome composition and maintenance of intestinal homeostasis.^70^ NLRP6 inflammasome signaling is co-modulated by microbiota-derived metabolites, which regulates epithelial IL-18 secretion and AMP expression profiles.^71^ Moreover, the NLRP6 inflammasome governs intestinal ‘sentinel’ goblet cell mucus secretion, which offers critical protection against pathogens.^72,73^ Beyond its role with regard to the bacterial kingdom, NLRP6 regulates intestinal antiviral innate immunity.^74^ Importantly, the impact of NLRP6 on microbiota community structure is dependent on the background microbiota in the vivarium, with dysbiosis occurring in mice lacking NLRP6 only in the presence of distinct microbiome configuration containing pathobionts such as *Helicobacter spp*.^75^ Another notable example of NLR assembling inflammasomes is NLRP3. Regulation of NRLP3 inflammasome signaling is required to maintain intestinal homeostasis. In patients with ulcerative colitis, a surplus of anti-commensal IgG engages gut-resident FcγR-expressing macrophages, inducing NLRP3- and reactive oxygen species-dependent production of the pro-inflammatory cytokine IL-1β.^76^ Upon intestinal injury, certain members of the microbiota such as *Proteus mirabilis* stimulate monocytes to induce NLRP3-dependent IL-1β release, which elicits intestinal inflammation.^77^ Moreover, sensing of intact bacterial peptidoglycan and peptidoglycan fragments by the innate immune system through numerous PRRs is necessary for proper development of immune cells and other tissues (reviewed in^78^). Another crucial PRR interacting with the microbiota through inflammasome signaling is the absent in melanoma 2 (AIM2). The AIM2 inflammasome was described to regulate intestinal homeostasis through the IL-18/IL-22/STAT3 pathway.^79^ Mammalian peptidoglycan recognition proteins (PGRPs) protect the host from colitis by promoting balanced microbiota configuration and by preventing production of IFNγ by NK cells in response to injury.^80^ These protective effects are in part achieved synergistically with NOD2.^81^ IPAF is an important member of the NOD‐LRR family of proteins. It recognizes intracellular flagellin and activates inflammasomes, stimulates caspase‐1, and promotes IL‐1β production in a TLR5‐independent manner in *Salmonella*-infected macrophages.^82^ However, its role in host-commensal interplay is still not clearly defined. Other PRRs potentially implicated in regulating host-microbiome symbiosis requiring further exploration are RIG-I-Like Receptors (RLRs)^83^ and OAS-Like Receptors (OLRs).^84^

An underappreciated area of microbiota research is represented by commensal protists. In an elegant study on transkingdom interactions, the authors demonstrate that the murine commensal protist *Trichomonas musculis* protects against enteric bacterial infection by activating epithelial inflammasome signaling, and thus promoting DC-driven Th1 and Th17 immunity.^85^

Monocytes and macrophages are crucial innate immune effector cells and have vital homeostatic roles.^86^ Recent research started to shed light on the relationships between these monocytes/macrophages and the commensal microbiota. A large microbiota-derived polysaccharide has been shown to induce an anti-inflammatory gene signature in murine intestinal macrophages.^87^ Moreover, butyrate can drive monocyte-to-macrophage differentiation through histone deacetylase 3 (HDAC3) inhibition, thereby amplifying antimicrobial host defense.^88^ Recently, it has been demonstrated that a soluble microbiome-derived metabolite, trimethylamine N-oxide (TMAO), can drive murine macrophage polarization in an NLRP3 inflammasome-dependent manner.^89^

Innate lymphoid cells (ILCs) are a more recently discovered heterogenous innate immune cell population specialized in the rapid secretion of polarized cytokines and chemokines to combat infection and promote mucosal tissue repair.^90^ ILCs have been categorized into three distinct types based on transcription factors and cytokine signatures. However, an in-depth single-cell transcriptome and chromatin state profiling hints towards a much more diverse landscape of ILCs.^91^ ILCs represent a rapidly growing new research area reviewed more comprehensively elsewhere.^92,93^ The phenotypic diversity and functional plasticity of the host’s intestinal ILCs are shaped by integrating signals from the microbiome.^91^ One factor regulating proliferation and function of group 3 ILCs is the microbial metabolite sensor Ffar2.^94^ Recently, a dichotomous regulation of group 3 ILCs by a pair of *Helicobacter* species in mice was identified. These species activated ILCs but negatively regulated the proliferation of group 3 RORγt^+^ ILCs that are crucial for host immunity and inflammation.^95^ Type 3 ILCs mediate immune surveillance of microbiota configuration to facilitate early colonization resistance through a transcriptional regulator ID2-dependent regulation of IL-22.^96^ NCR^+^ ILC3 cells were demonstrated to be essential for maintaining cecal homeostasis in mice during *Citrobacter rodentium* infection.^97^ A commensal linked with risk for allergic disease in children, *Ruminococcus gnavus*, induces infiltration of the colon and lung parenchyma by eosinophils and mast cells in mice via a cascade implicating type 2 ILCs, hinting at a crucial role of ILCs in immune tolerance.^98^

### Interactions between the adaptive immune system and the microbiota

In addition to the impacts of host-microbiota interactions on innate immune function, recent research also uncovered mechanisms governing mutualism between the microbiome and the adaptive immune system (Fig. 1). One example involves B cells, crucial mediators of gut homeostasis by producing a large array of secretory IgA antibodies responsive to commensals.^46^ Several grams of IgA are secreted every day in the human intestines.^99^ Secretory IgA can be produced either in a T cell-independent or a T cell-dependent manner. IgA produced in a T cell-dependent way plays a more important role in shaping gut microbial communities.^100^ The relationship between intestinal IgA and microbiota is mutualistic, in that a diversified and selected IgA repertoire contributes to maintenance of a diversified and balanced microbiome, which facilitates the expansion of Foxp3^+^ regulatory T cells sustaining homeostatic IgA responses in a regulatory loop.^101^ Interestingly, intestinal secretory IgA antibodies preferentially coat colitogenic bacteria, therefore preventing perturbation of enteric homeostasis and inflammation.^102^ In the absence of B cells, or of IgA, intestinal epithelia upregulate epithelium-inherent immune defense mechanisms mediated by interferon-inducible response pathways, which are associated with subsequent changes in microbiome composition. Interestingly, the simultaneous repression of Gata4-related metabolic functions in this scenario results in impaired intestinal absorption and metabolic alterations.^103^ Recently, a new subset of subepithelial mesenchymal cells expressing the cytokine RANKL were identified to serve as intestinal M cell inducers, thereby fostering IgA production and gut microbiota diversification.^104^

Studies conducted during the past decade provided a more detailed picture of the crosstalk between the gut microbiome and CD4^+^ regulatory T cells. A subset of colonic regulatory CD4^+^ T cells lack differentiation in GF mice resulting from the absence of bacterial consortia capable of fermenting dietary fiber into short-chain fatty acids (SCFAs).^105–107^ Reactivity to intestinal bacteria seems to be a 'healthy' property of both intestinal and systemic human CD4^+^ T cells, which may support homeostasis by providing a large pool of immune cells protective against pathogens.^108^ Of these cells, the Th17 subset is intensely studied because of its ambiguous roles in both host protection and inflammatory disorders.^109^ The intestine harbors functionally distinct Th17 cell populations and their inflammatory propensity is largely determined by distinct bacteria eliciting their differentiation. Th17 cells elicited by SFB are non-inflammatory, while Th17 cells induced by *Citrobacter* are a potent source of inflammatory cytokines.^110^ While it is well established that microbiota is involved in Th17 differentiation in the intestine^36^ and the skin^111^, oral barrier Th17 cell development seems to be largely independent from microbial colonization.^112^ Another example of microbiome regulation of adoptive T cell responses involves CD8^+^ (cytotoxic) T cells, whose effector functions are paramount in elimination of intracellular pathogens and cancer cells. While these cells require priming by professional antigen-presenting cells (APCs) and are amplified by CD4^+^ T cell signaling^113^, antigen-activated CD8^+^ T cells show no transition into memory cells in GF mice, as microbiota-derived SCFAs are required to promote their memory potential.^114^ A fraction of primary bile acids secreted into the intestine escape into the colon where they are converted into secondary bile acids by the microbiota, and may have various signaling functions that are yet to be fully explored. A recent work showed that microbiota-derived secondary bile acids regulate gut RORγ^+^ regulatory T cell homeostasis.^115^

Follicular helper T (Tfh) cells are specialized to assist B cells, and are crucial for germinal center formation, affinity maturation, and generation of high-affinity antibody responses and memory B cells.^116^ Tfh cells are implicated in maintenance of microbiota homeostasis as highlighted by studies showing that impairment of Tfh cells resulting from lack of expression of co-receptor programmed cell death 1 (PD-1) or ATP-gated ionotropic P2RX7 receptor can alter gut microbiota composition.^117,118^ The relationship between Tfh cells and the microbiota is reciprocal, as Tfh cell differentiation is impaired in GF mice and can be restored by administration of Toll-like receptor 2 (TLR2) agonists that activate T cell-intrinsic MyD88 signaling.^119^ In mice, SFB can induce Tfh cell differentiation in Peyer’s patches by limiting the access of IL-2 to CD4^+^ T cells, thereby amplifying the master regulator Bcl-6 of Tfh cells.^120^ The microbiota-Tfh axis may also be relevant in autoimmune diseases, as in mice SFB-induced Tfh cell differentiation can boost autoantibody production and thus exacerbate arthritis.^120^

Additionally, recent studies began to uncover the relationships between the microbiota and tissue-resident DCs, which represent an important class of APCs shaping immune responses. DCs are able to send their dendrites outside the epithelium to directly capture bacteria.^121^ Recently, a Syk kinase-coupled signaling pathway in DCs was described to be critical for microbiota-induced production of IL-17 and IL-22 by CD4^+^ T cells.^122^ Moreover, a noncanonical NF-κB-inducing kinase (NIK) was recently reported to be a crucial mediator of mucosal DC function. In the same study, DC-specific NIK altered enteric IgA secretion and microbiota homeostasis, rendering mice vulnerable to enteric pathogens.^123^

A relatively unexplored set of immune cells with crucial relationship to the commensal microbiota is represented by invariant natural killer T cells (iNKTs). The gut microbiota affects the phenotypes and functions of iNKTs in mice, with iNKTs from GF animals showing a less mature phenotype and decreased activation by antigens.^124^ Mono-colonization of neonatal GF mice with the commensal *Bacteroides fragilis* or exposure to a purified sphingolipid originating from *B. fragilis* was able to restore iNKT cell numbers in GF mice and to protect the animals from oxazolone-induced colitis.^125^

## Influence of environmental microbiome perturbation on the immune system

The gut microbiome is shaped by a wealth of environmental factors whose impacts dominate over host genetics.^126^ These environmental factors, including diet, antibiotic use, westernized lifestyle, etc., are potential triggers of inflammatory and autoimmune diseases.^127^ Understanding of environmental gut microbiome modulation and its impact on disease propensity is still in its infancy. Currently, the best-studied environmental sources of microbiome variation are antibiotic treatment and diet.

### Antibiotic-induced microbiome disturbances

Antibiotics are an indispensable treatment against infectious diseases and their introduction has dramatically changed healthcare and human life expectancy. However, evidence suggests that antibiotic use during childhood is associated with the development of a range of immune-mediated diseases, including allergies and IBD.^21,128^ Intake of antibiotics profoundly affects the composition and function of the gut microbiota, and may introduce long-lasting adverse effects on the host.^129^ Different immune cell subsets and functions can be altered by antibiotic-driven gut microbial dysbiosis. In rats, administration of antibiotics inhibits intestinal mucosal mast cell activation and suppresses dietary lipid uptake.^130^ Broad-spectrum antibiotic-mediated microbial perturbation and depletion of microbiota-derived SCFAs causes hyperactivation of intestinal macrophages and expansion of proinflammatory T helper cells and increases susceptibility to infection.^131^ Furthermore, antibiotic treatment permits overgrowth of enteric fungi, thereby promoting pulmonary M2 macrophage polarization, which in turn promotes allergic airway inflammation.^132^ Microbiota disruption by antibiotics results in enhanced pathogen-specific Th1 cell responses and tissue pathology in an CX3CR1^+^ MNP-dependent manner.^133^ Significantly reduced RORγt^+^ Tregs in GF or antibiotic-treated mice promote Th2 type-associated immune responses and inflammation upon helminth infection.^134^ In humans with pre-existing immune system impairment, microbiome depletion through broad-spectrum antibiotics not only results in a diminished antibody response to seasonal influenza vaccination, but also leads to augmented circulatory inflammatory signatures and altered plasma metabolome profiles.^135^ The long-term health consequences of antibiotic-induced microbiome alterations in humans merit more long-term observational studies and clinical trials.

### Diet-induced microbiome alterations

Recent studies began to unravel the links between dietary microbiota modulation and host immunity. Western style diets profoundly affect gut microbiome configuration and adversely impact on host immunity.^136^ For example, a diet high in saturated fats increases the levels of taurocholic acid, a secondary bile acid, and in turn fosters the expansion of *Bilophila wadsworthia*. This pathobiont promotes Th1 type immune responses and increases susceptibility to colitis in *IL10*^*–/–*^ mice.^137^ High-fat diet can also aggravate disease severity in chemically induced murine colitis by disturbing the homeostasis of intestinal DCs, possibly by reducing butyrate and retinoic acid levels.^138^ Dietary long-chain fatty acids may exacerbate autoimmunity in the central nervous system (CNS) by modulating the gut microbiome and metabolome.^139^ In mice, intake of dietary carbohydrates,^105^ certain probiotics,^140^ artificial sweeteners^141^ and emulsifiers^142^ can modulate host immunity and inflammation, in part mediated by compositional changes of the gut microbiome. In humans, individuals with higher fecal abundance of the bacterial genus *Dialister* and lower levels of *Coriobacteriaceae* family members show reduced serum levels of the pro-inflammatory cytokine IL-6 after short-term consumption of whole grains.^143^

In addition to dietary quantity and content, the timing of dietary intake has been recently shown to affect microbiome composition and in turn immunity. Intermittent fasting ameliorates disease severity in a murine model of autoimmune encephalomyelitis and in patients with multiple sclerosis by microbiota-mediated balancing of IL-17-producing and regulatory T cells.^144^ In a murine colitis model, a fasting-mimicking diet exerted a protective effect through modulation of the gut microbiome including an increase of *Lactobacillus*.^145^ In contrast, mistimed dietary intake accelerates alcohol-associated colonic carcinogenesis by reducing the number of butyrate- and SCFA-producing bacteria, which causes mucosal Th17/regulatory T cell imbalance.^146^

Of note, the impact of the microbiome on immunity in laboratory mice can be vastly divergent from that in humans, which is in part explained by differences in microbiota between mice raised in laboratory versus wild environments. Mice with a natural wild microbiota are more resilient to environmental challenges and show responses to immunotherapy that are more resemblant of  humans.^147^ Therefore, it is important to study the impact of environmental exposures on the host immune system in a context of such human-like microbiota configuration, which may promote better understanding of immune system-microbiota interactions and their translation into clinical applications.

## Dysregulation of microbiome-immunity interaction in disease

**Fig. 2:**
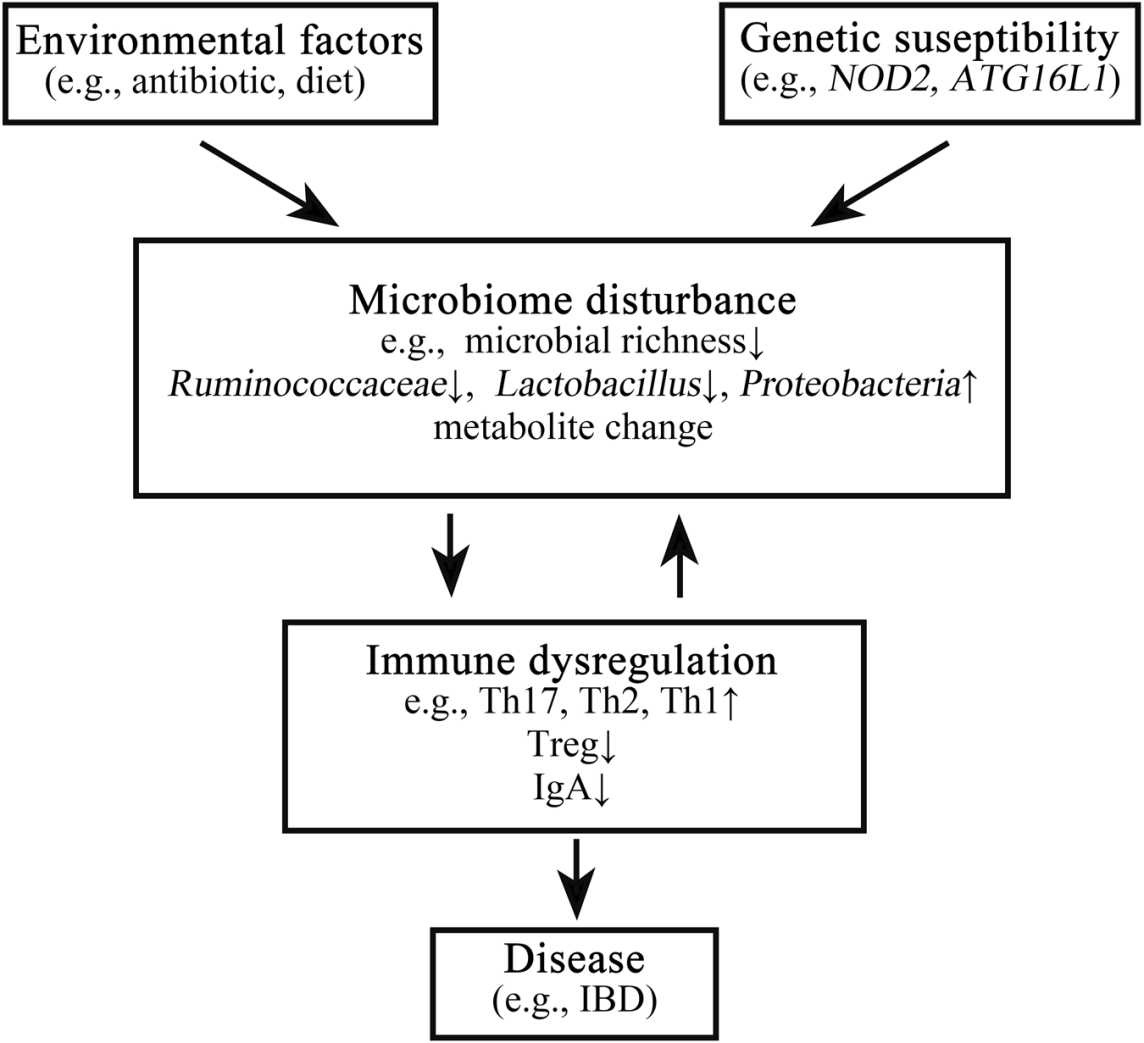
Dysregulation of microbiome-immunity interaction in disease. Under the influence of certain environmental factors and host genetic susceptibility, aberrant interactions between the microbiome and the host’s immune system contribute to the development of various immune-mediated disorders. In IBD as an example, antibiotic use or dietary changes, in the presence of genetic susceptibility (e.g., *NOD2* mutation), may lead to alterations of the gut microbiome configuration, including decreased richness and perturbed taxonomic and metabolite composition. These microbiome alterations are strongly associated with aberrant mucosal immune responses, including upregulated Th17, Th1 and Th2 type responses, downregulated T regulatory cells, and dysregulated humoral immunity. This may finally result in chronic, clinically-overt intestinal inflammation and tissue injury.

Aberrant interactions between the microbiome and the host’s immune system in genetically susceptible individuals may contribute to the development of complex immune-mediated diseases (Fig. 2). Among these, the most extensively studied examples include IBD, systemic autoimmune diseases, cardiometabolic diseases and cancer. Additionally, the microbiome-immunity link has been suggested to modulate other ‘multi-factorial’ diseases (e.g., neurodegenerative diseases) but requires further human studies. More importantly, the causal effect of the microbiome on immune dysregulation in most human disorders listed above remains to be proven.

### Inflammatory bowel disease

IBD, mainly encompassing Crohn’s disease (CD) and ulcerative colitis, is a chronic, recurrent inflammatory disorder of the gastrointestinal tract, characterized by a growing global prevalence.^148^ Multiple lines of evidence point towards central roles of gut microbiome perturbations in the pathogenesis of IBD. These include a reduced bacterial diversity and marked shifts in abundance of certain bacterial taxa, including decreased abundance of *Bacteroides*, *Firmicutes*, Clostridia, *Lactobacillus*, *Ruminococcaceae* and increased abundance of *Gammaproteobacteria* and *Enterobacteriaceae*,^149,150^, coupled with altered microbiome-associated metabolite profiles.^151,152^ The breakdown of the tightly regulated intestinal barrier leads to translocation of bacterial symbionts into the mucosal layer, fueling aberrant host immune responses and tissue injury.^153^ As such, disruptions of gut barrier integrity, including the mucus layer, epithelial cell junctions, and AMP secretion are all believed to be involved in IBD pathogenesis.^154^ For example, mice deficient in Muc2 may develop spontaneous colitis,^155^ and mucus layer defects due to Muc2 mutation drive early gut dysbiosis in colitis-prone mice.^156^

Genome-wide association studies revealed so far more than 200 susceptibility loci for IBD, many of which encode proteins involved in innate and adaptive immune sensing and response to bacterial signals. Among these, mutation in the *NOD2* gene was the first to be confirmed to be strongly associated with susceptibility to CD.^157,158^ NOD2 is an intracellular PRR capable of recognizing bacterial peptidoglycan-conserved motifs. NOD2 acts as a critical regulator of the intestinal commensal microbiota, by controlling the expression and secretion of AMPs^159^ (see above) and suppressing the expansion of certain proinflammatory bacterial species such as *Bacteroides vulgatus*.^64^ The dysregulated microbiome-immunity interaction in the context of *NOD2* mutation is assumed to play important roles in CD pathogenesis. Likewise, mutations in autophagy-related 16-like 1 (*ATG16L1*), another CD-associated risk allele, not only result in impaired exocytosis in Paneth cells,^160^ but potentiate inflammatory responses and necrosis of intestinal epithelial cells through modulation of IL-22 signaling.^161^ The role of inflammasome signaling in regulating the crosstalk between the microbiome and immunity is likewise implicated in pre-clinical IBD models. For example, perturbation of the NLRP6 inflammasome pathway results in susceptibility to murine colitis through expansion of members of the Prevotellaceae family in some vivaria,^70^ and promotes intestinal inflammation in *IL10*^*–/–*^ mice by enhancing colonization with *Akkermansia muciniphila*.^162^ The contribution of adaptive immune responses to the expansion of IBD-associated pathobionts, including aberrant roles of effector T cells, regulatory T cells and antibody-mediated humoral immunity, has been reviewed extensively elsewhere.^153^

Notwithstanding all of these data, whether microbiome alterations represent the cause or consequence of intestinal inflammation remains unclarified to date. Some emerging evidence supports a causal role of gut dysbiosis in IBD, since transfer of disease-associated microbiota triggers CD-like inflammation in genetically susceptible GF recipient mice.^163^ Microbiota from IBD patients transplanted to GF mice likewise induces imbalances in intestinal Th17 and RORgt^+^ regulatory T cells.^164^ More strikingly, one single pathobiont, *Mucispirillum schaedleri*, was demonstrated to be sufficient to trigger a Th1 cell-driven intestinal inflammation in mice deficient in both NOD2 and CYBB.^165^ Similarly, ectopic colonization of oral *Klebsiella spp*. derived from IBD patients, induces Th1-type intestinal inflammation in *IL10*^*–/–*^ mice.^166^ Furthermore, abnormal T cell and B cell adaptive immunity can be transmitted to GF mice from infant-harbored microbiome born to IBD-prone mothers.^167^ Increasing knowledge on molecular impacts of distinct commensals and their small-molecule products on the clinical features of IBD may enable the development of future targeted interventions.

### Rheumatoid arthritis

Rheumatoid arthritis (RA) is a systemic autoimmune disorder mainly involving the joints, characterized by synovial inflammation and bone cartilage destruction. The pathogenesis of this highly debilitating disease is currently unclear. Genetic (e.g., HLA-DRB1), microbiome and environmental factors have been implicated in the pathogenesis of RA. An increased abundance of *Prevotella copri* was reported in treatment-naïve new-onset RA patients^168,169^ and in individuals at high risk for RA.^170^ Another study identified a strong link between three rare genera (*Collinsella*, *Eggerthella* and *Faecalibacterium*) and RA, among which *Collinsella* is associated with proinflammatory IL-17A production.^171^ In a Chinese cohort, RA patients displayed an over-representation of *Lactobacillus salivarius* and reduced levels of *Haemophilus spp*. in intestinal, dental and saliva specimens.^172^ Microbiome-derived metabolites, most notably SCFAs, interact with a variety of immune pathways implicated in RA.^173^ Spontaneous development of T cell-mediated autoimmune arthritis in *IL1rn*^*–/–*^ mice requires the activation of TLR2 and TLR4 by microbial ligands.^174^ Dysbiotic microbiota from *IL1rn*^*–/–*^ mice elicits a IL17 response by intestinal lymphocytes.^175^ Moreover, genetically susceptible mice colonized with dysbiotic microbiota from RA patients show an enhanced Th17 type response.^169^ Similarly, inoculation of SFB into GF mice is sufficient to induce Th17 activation and to instigate autoimmune arthritis.^176^ In addition to the enteric bacteria, the periodontal pathobiont *Porphyromonas gingivalis* can induce a TLR2- and IL-1-mediated Th17 response and thereby exacerbate autoimmune arthritis.^177^ Future studies are required to determine the influence of RA treatment on the microbiome and the causal role of microbiome alterations potentially modulating human RA.

### Cardiometabolic disease

Chronic low-grade inflammation is considered a hallmark of metabolic disorders, including diabetes mellitus, obesity, atherosclerosis and non-alcoholic fatty liver disease (NAFLD). In metabolically highly active organs such as the liver or adipose tissue, the crosstalk between immune cells and parenchymal cells plays a critical role in the pathogenesis of metabolic diseases.^178^ Growing evidence shows that gut microbiome-derived metabolites can reach systemic circulation through the gut barrier and fuel metabolic inflammation.^179^ Various TLRs in the liver recognize bacterial ligands and trigger downstream inflammatory cascades. Activation of these TLRs can contribute to the development of NAFLD and nonalcoholic steatohepatitis (NASH), with the most extensively studied pathway being LPS-TLR4 signaling.^180^ In addition to TLRs, the NLRP6 and NLRP3 inflammasomes may exert protective effects against NAFLD/NASH through modulation of the gut microbiota.^181^ Multiple interactions between the host’s immune system and the gut microbiota were reported to be involved in type 1 diabetes (T1D). For example, GF non-obese diabetic mice lacking MyD88 signaling robustly develop T1D, while microbial colonization of these mice attenuates the disease.^56^ Depletion of *Akkermansia muciniphila* causes systemic translocation of endotoxin-activated CCR2^+^ monocytes. These in turn activate innate pancreatic B1a cells, resulting in increased insulin resistance.^182^ Furthermore, the crosstalk between the microbiome and immunity plays a crucial role in obesity. For example, microbiome-derived tryptophan metabolites modulate white adipose tissue inflammation in obesity, mediated through the miR-181 family of microRNAs.^183^ Recently, the innate immune sensor NLRP12 was shown to decrease high fat diet-induced obesity in mice by preserving SCFA-producing members of the *Lachnospiraceae* family.^184^ One of the most perilous common sequelae of cardiometabolic disease is atherosclerosis and its complications. The gut microbiota-derived metabolite TMAO has been linked to atherosclerotic heart disease in both mice and humans.^185^ Interestingly, TMAO augments arthrosclerosis by upregulating the macrophage scavenger receptors CD36 and SR-A1, and by reinforcing cholesterol accumulation in macrophages and foam cell formation.^186^

### Cancer

Interactions between the gut microbiota and the immune system are believed to impact on cancer immune surveillance. In the context of colon cancer, NK cell killing of tumors is directly inhibited by the presence of *Fusobacterium nucleatum* in the tumor microenvironment. This is in part mediated by binding of the bacterium’s Fap2 protein to the human TIGIT receptor.^187^ Higher amounts of *F. nucleatum* in human colorectal cancer tissue are furthermore associated with a lower density of CD3^+^ T cells, a population associated with a more favorable clinical outcome.^188^ In remote tissues such as the liver, the intestinal commensal *Clostridium* species utilize bile acids as messengers to enhance the antitumoral effect of hepatic CXCR6^+^ NKT cells, affecting both primary and metastatic liver tumors.^189^ The microbiome has been recently suggested to also modulate anticancer immunotherapy responses. For example, higher abundances of the commensals *Bifidobacterium longum*, *Collinsella aerofaciens*, and *Enterococcus faecium* stimulate a more favorable T cell-mediated response to anti-PD-1 therapy in both preclinical models and patients suffering from metastasized melanoma.^190–192^ Another study revealed a positive correlation between fecal *Akkermansia muciniphila* abundance and PD-1 blockade efficacy in patients with epithelial tumors, potentially dependent on CCR9^+^CXCR3^+^CD4^+^ T lymphocyte recruitment and IL-12 secretion.^193^ Immune responses to other anticancer treatments, including CTLA-4 blockade^194^ and cyclophosphamide,^195^ were also associated with distinct gut microbiome configurations. Unraveling the role of the gut microbiome in anticancer immune surveillance and immunotherapy may hold great promise in optimizing treatment responses in cancer patients, and has been reviewed elsewhere in greater detail.^13,196^

Aside from the gut microbiome, most recent research begins to explore the role of intra-tumor tissue microbiome in regulating cancer immunity. For example, intra-tumor microbiota in pancreatic adenocarcinoma (PDAC) in mice and humans promotes carcinogenesis through induction of a tolerogenic immune program, including suppressive differentiation in monocytes via selective TLRs and T cell anergy.^197^ In addition, the presence of *Gammaproteobacteria* in murine colon cancer and human PDAC contributes to resistance against therapy with gemcitabine.^198^ Interestingly, the intra-tumor microbiome in long-term survivors of PDAC patients exhibits higher microbial diversity, which may induce potent immune infiltration and antitumor immunity.^199^ These studies indicate the potential of tumor tissue-resident microbiota as a therapeutic target, which warrants further mechanistic studies.

## Crosstalk between microbiota and extra-intestinal organ immunity

**Fig. 3:**
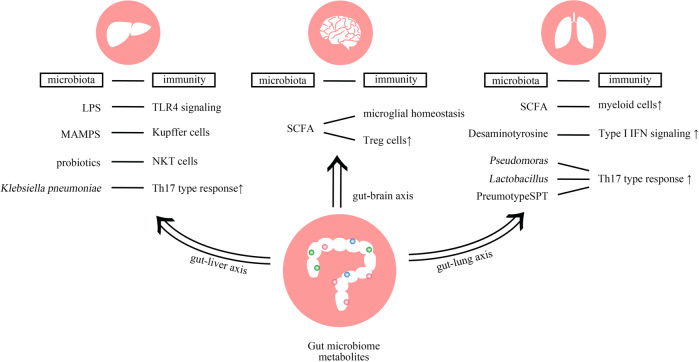
Microbiome-immunity interaction in extra-intestinal organs. The gut microbiome and microbiome-associated metabolites translocate from the intestinal lumen to various organs (e.g., liver, brain or lung) through the circulatory system, and subsequently induce tissue-specific local immune responses. In the liver, bacterial LPS is recognized by TLR4 in different cell types, leading to upregulation of various pro-inflammatory chemokines and adhesion molecules. MAMPs influence the number, function and maturation of Kupffer cells, and glycolipid antigen-containing probiotics can activate hepatic NKT cells. The gut-resident pathobiont *Klebsiella pneumoniae* can translocate and induce Th17 cell responses in the liver. In the CNS, microbiome-derived SCFAs regulate microglial homeostasis, and promote regulatory T cells to counter-regulate CNS autoimmunity. In the lung, SCFA-induced primed myeloid cells translocate to the lung and shape the pulmonary immunological landscape. *Clostridium orbiscindens*-derived product desaminotyrosine modulates type I IFN signaling. In addition, exposure to different lung-resident microbes (e.g., *Pseudomonas*, *Lactobacillus*, pneumotypeSPT) is associated with an enhanced Th17 type response.

Although most studies in the field to date focused on the interplay of microbiota and mucosal immunity in the intestine, interactions of both the gut microbiota and extra-intestinal microbiota communities with extra-intestinal organ immunity have been gaining increased attention (Fig. 3). Emerging evidence highlights that the local microbiomes of extra-intestinal mucosal surfaces provide niche-specific functions, including modulation of organ-specific immune responses.

### Skin

Alike the intestine, the skin (the body’s largest organ) represents a dynamic and complex ecosystem, harboring and interacting with a plethora of locally-entrenched commensal microorganisms. High throughput sequencing-based studies revealed a diversity of site-specific but temporally stable microbial communities in the healthy human skin^200,201^ featuring inter-individual variability.^202^ The skin microbiota induces protective and regulatory immunity that contributes to host-microbe mutualism. Skin-resident commensals not only effectively control the equilibrium of T effector and regulatory T cells in the tissue, dependent of IL-1 and MyD88 signaling,^111^ but also regulate components of the cutaneous complement system^203^ as well as the expression of various cutaneous AMPs.^204^ Certain aspects of the regulation of cutaneous innate and adaptive immunity by the skin microbiome feature strain specificity. One of the most highly abundant skin commensals, *Staphylococcus epidermidis*, can specifically induce homing of CD8^+^ T cells primed by CD103^+^ DCs into the epidermis and can promote skin antimicrobial responses in an IL17-dependent manner.^205^ Furthermore, the *S. epidermidis*-specific CD8^+^ T cell response is restricted to non-classical MHC class I molecules, which also promote tissue repair.^206^ During skin injury,TLR2 recognition of *S. epidermidis* cell wall component lipoteichoic acid suppresses skin inflammation and inhibits release of inflammatory cytokines, thereby promoting wound healing.^207^ It should be noted that colonization with skin commensal during the neonatal period is crucial for establishing immune tolerance through massive accumulation of active T regulatory cells in the neonatal skin, collaboratively driven by hair follicle morphogenesis.^208,209^ Moreover, epidermal keratinocytes also actively participate in cutaneous immune defenses. Microbial metabolites, such as SCFAs produced by the commensal skin bacterium *Propionibacterium acnes*, can modulate keratinocyte inflammatory activity through inhibition of the keratinocytes’ histone deacetylases.^210^ Furthermore, cutaneous commensals such as coagulase-negative *Staphylococcus* strains produce antimicrobials that protect from pathobionts such as *Staphylococcus aureus*.^211^

Skin dysbiosis has been associated with different inflammatory skin disorders, including atopic dermatitis^212^ and psoriasis.^213^ Whether skin dysbiosis is the cause or consequence of these disorders is not yet clarified, but it has been proposed that locally amplified immune responses to particular skin microbes, or increased microbial load, in the setting of impaired skin barrier and genetic predisposition, might contribute to pathology.^214^ For example, skin colonization with *Staphylococcus aureus* promotes skin allergy in a mouse model of atopic dermatitis through δ-Toxin-induced mast cell activation.^215^ Furthermore, epidermal JunB is critical for immune-microbiota interactions, as mice lacking JunB expression in skin epithelial cells are characterized by augmented Th2 and Th17 type immune responses, accompanied by increased *S. aureus* colonization.^216^ However, many open questions remain to be explored, including the molecular basis of cutaneous microbiota-immune interactions and mechanisms by which the cutaneous immune system discriminates between skin commensals and pathogens.

### Lung

Emerging evidence highlights an important crosstalk between the gut microbiome and the lung (‘gut-lung axis’). Alterations in the gut microbiome or microbiome-derived metabolites may impact on lung immunity in the context of pulmonary diseases. Gut commensals regulate antiviral immunity at the respiratory mucosa through inflammasome activation upon influenza A virus infection.^217^ Accordingly, GF mice show an impaired pulmonary pathogen clearance.^218^ Microbiome-derived SCFAs promote bone marrow hematopoiesis, and the primed myeloid cells subsequently migrate to the lung, shaping the lung’s immunological landscape and conferring protection against airway inflammation.^219^ Desaminotyrosine, a product derived from the gut commensal *Clostridium orbiscindens*, exerts distal effects on the lung to protect against influenza through modulation of type I IFN signaling.^220^

Additionally, recent evidence points towards a potential of a locally entrenched lung microbiota possibly impacting pulmonary immunity.^221^ In mice, the rapid formation of an airway microbiome within the first 2 postnatal weeks is critical for immune tolerance to inhaled allergens through PD-L1-related mechanisms.^222^ The human microbiome in the lower respiratory tract forms within the first 2 postnatal months, alongside lung immune maturation.^223^ Alterations of the lung microbiota has been implicated in exacerbation of chronic pulmonary diseases, including chronic obstructive pulmonary disease, asthma and cystic fibrosis.^224^ Notably, exposure to different lung microbes is associated with different cellular immune responses. For example, enrichment of *Pseudomonas* and *Lactobacillus* in mouse models of chronic lung inflammation,^225^ or pneumotypeSPT derived from a diseased human bronchoalveolar system,^226^ is related to an enhanced Th17 type response. Pathobionts such as members of *Proteobacteria* induce severe TLR2-independent airway inflammation and lung immunopathology.^227^ More recent evidence suggests that certain lung commensals may instigate the development of pulmonary adenocarcinoma by activating γδ T cells that produce IL17. This highlights the putative role of a lung microbiome-immunity crosstalk in lung cancer.^228^ However, the study of the lung microbiome and the interplay between commensal microbial communities and pulmonary immunity is only in its infancy, with many more mechanistic insights expected to be revealed in future studies.

### Liver

The liver features direct anatomical connection to the gastrointestinal tract via the portal venous circulation and bile duct system, thereby being constantly exposed to bacterial products of gut microbiome origin (‘gut-liver axis’). Intestinal commensals and their products were repeatedly reported to translocate from the intestinal lumen to the liver in certain contexts, in which they may impact hepatic immune responses. For example, microbial-associated molecular patterns (MAMPs) from gut bacteria can directly influence the number, function and maturation of hepatic Kupffer cells (KCs), a critical componentof the hepatic innate immune system.^229^ Intestinal pathogens may exacerbate immunological hepatic injury by activating DCs and NKT cells in the liver.^230^ Similarly, glycolipid antigen-containing probiotics were reported to stimulate hepatic NKT cells in a strain- and dose-dependent manner.^231^ Hepatic stellate cells, the main fibrosis-inducing cell line in the liver, can also be directly stimulated by bacterial lipopolysaccharide (LPS), mainly through induction of TLR4 signaling. This results in an upregulation of multiple chemokines and adhesion molecules.^232^ Innate immune sensing of gut-derived microbial products by different TLRs, including TLR4, TLR9, TLR5, and their downstream impacts on liver inflammation in the context of NAFLD/NASH have been recently reviewed elsewhere.^180^

Liver inflammation impacted by gut microbiota was also described in primary sclerosing cholangitis (PSC), a chronic inflammatory and cholestatic liver disease. The enteric pathobiont *Klebsiella pneumonia* cultured from PSC patient specimens was demonstrated to damage the intestinal epithelial barrier, thereby inducing bacterial translocation that promotes Th17 cell responses in the murine liver.^233^ Interestingly, a recent study showed alterations of the bile microbiota in PSC patients, characterized by reduced biodiversity, higher abundance of the pathobiont *Enterococcus faecalis*, and increased levels of the noxious secondary bile acid taurolithocholic acid.^234^ However, it remains unclear whether these alterations are causally involved in PSC or are merely a consequence of biliary disease.

Recent studies also demonstrated carcinogenic effects of microbiome-derived small molecules via regulation of immune responses in liver malignancy, including secondary bile acid mediating upregulation of hepatic NKT cells,^189^ deoxycholic acid modulating the inflammatory secretome,^235^ lipoteichoic acid regulating prostaglandin E2 expression,^236^ and LPS signaling through TLR4.^237^

### Central nervous system

The development of a healthy brain and balanced neuro-immunity relies on integration of numerous endogenous and environmental cues. Among these, molecular signals originating from the gut microbiome may play prominent roles in modulating brain cell function.^238^ Microglia are among the primary innate immune cells in the CNS, and are instrumental in CNS immune defense and contribute to brain development and homeostasis.^239^ The microbiota contributes to microglia homeostasis, potentially mediated by signaling through SCFAs.^240^ GF mice display marked defects in microglia structure and function and hence feature impaired CNS innate immune responses.^240,241^ Interestingly, the maternal microbiome impacts on microglial development during prenatal stages, and microglial perturbations associated with the absence of microbiota manifest in a sex-dimorphic manner.^242^ Both microbial dysbiosis and microglial dysfunction have been described in several neurological diseases, including behavioral, inflammatory and neurodegenerative disorders.^243^ Whether microbiota-microglia interactions contribute to the pathogenesis of these disorders merits further studies.

Moreover, diet-derived SCFAs were reported to promote regulatory T cells to counter-regulate autoimmunity in the CNS,^139^ and the intestinal microbiota modulates meningeal IL-17^+^ γδ T cells, which impact on the pathogenesis of ischemic brain injury.^244^ Despite tremendous recent advances, the study of the interplay between the microbiome and neuro-immunity in health and disease is still in its infancy. Some studies shed light on possible mechanisms driving such putative 'gut-brain axis' in the context of neuro-immunity. For example, depletion of gut commensal bacteria by antibiotic treatment dampens the progression of experimental autoimmune encephalomyelitis in mice, which is suggested to be mediated by induction of IL-10-producing regulatory T cells.^245^ Offsprings of pregnant female mice that harbor certain gut bacteria with a propensity to induce T helper 17 response are at increased risk of developing neurodevelopmental disorders.^246^ Interestingly in a murine maternal immune activation model, IL-17a-mediated inflammatory responses were shown to exert beneficial roles in improving social behaviors in offsprings of adult mice.^247^ Potential microbiota involvement in these mechanisms merits further studies. Continued research efforts in this direction may hold great therapeutic promise in uncovering new regulatory pathways impacting a variety of inflammatory, developmental and degenerative neurological diseases.

### Intra-organ low-biomass microbiomes

There is growing recent interest in utilizing next-generation sequencing to characterize sparsely populated low-biomass microbiomes in seemingly ‘sterile’ organs, such as the skin,^206^ lungs,^248^ reproductive organs^249^ and bile ducts.^234^ However, caution is required in interpreting such findings, as many studies that attempt to investigate low-biomass microbiome samples are challenged by high false positive signals resulting from contamination and sequencing-related challenges and artefacts.^250^ Contaminating microbial DNA may originate from multiple environmental sources, such as laboratory extraction, amplification and library preparation kits.^251^ Notably, the notion of the existence of a placental microbiome and its link to reproductive health was recently challenged by a thorough comparison of results using different kits, blank controls and complementary approaches of microbial detection not exclusively relying on sequencing.^252,253^ In order to avoid fallacious conclusions, strategies to control contamination must be considered when working with low microbial biomass tissues, including experimental and computational measures.^250,254–256^ Although promising, these strategies largely still await proof that signals uncovered from low-biomass microbiomes reliably translate into verifiable mechanistic biological insights.

## Challenges and pitfalls in immune-microbiome research

Recent research has greatly enhanced our understandings of the intimate but complicated crosstalk between the microbiome and the immune system. Nevertheless, many unknowns and challenges remain, in disentangling microbiome-immunity interactions  in homeostasis and disease.

Exploring the roles of the commensal microbiome in impacting immunity in health and in disease requires more mechanistic studies. Indeed, current evidence from animal models indicates a bidirectional relationship to exist between microbiome perturbation and immune dysregulation. As such, distinct microbiota and metabolites drive immune activation, and chronic inflammation conversely may shape the dysbiotic configuration and functions of microbial communities. However, a direct causal relationship between the microbiome and immunity before the onset or during early stages of disease has not been established in most medical conditions. Moreover, the role of other previously underappreciated microorganisms, including viruses, fungi, parasites and their impact on the host immunity, emerges as an important but challenging subject to be explored in future studies. As an example, while  recent research begins to uncover the role of fungi^257,258^ and viruses^259,260^ in IBD pathogenesis, the interplay between the mycobiome, virome and microbiome adds a layer of complexity in mining their impacts on innate and adaptive immune responses. Furthermore, many diseases of unknown etiology, including IBD, autoimmune arthritis and cancer, are influenced by both genetic and environmental factors (e.g., diet, smoking, etc.).^261^ It is imperative to investigate how the microbiome and the immune system interact in a context of environmental triggers and host genetics. Integration of multi-omics data sets, including metagenomics, single-cell transcriptomics, epigenomics, proteomics and metabolomics, will aid in elucidating how the gut microbiome and the immune system are cross-regulated in these differing and complex contexts. Importantly in all of these efforts, the microbiome research community massively uses laboratory mice that harbor a divergent microbiota from ‘wild’ animals and humans, thereby featuring a limited translational potential and reproducibility as compared to ‘real-life’ settings. The newly created ‘wilding mice’ with low genetic variability but a highly natural and resilient microbiota,^147^ may enable better mechanistic dissection of host-microbiome interactions and provide a valuable preclinical tool to phenocopy human immune responses. Indeed, a recent study has shown that the gut microbiota in wild mice can better recapitulate the natural phenotypes in humans, as laboratory mice receiving wild microbiota exhibit less susceptibility to influenza virus infection and colitis-induced tumorigenesis, which is associated with less infiltration of immune cells and enhanced anti-inflammatory responses.^262^ Future studies should consider incorporating similar approaches to better resemble natural microbiome-immune interplay in order to increase the translational potential of such studies.

In addition, many studies focusing on microbiome-immunity interaction have utilized 16S rRNA sequencing to characterize the microbiome, but this modality is limited by its genus-level and purely compositional resolution. Given that strain level resolution and functional insights are better served by shotgun metagenomic sequencing, the field is expected to increasingly rely on this more sophisticated methodology (in addition to metatranscriptomics, metabolomics, metaproteomics and culturomics) in decoding immune-microbiota interactions. Finally, the microbiome configuration and immune responses are both increasingly appreciated to be highly variable among human individuals, with more variances typically explained by inter-individual variation than by disease state. This inherent inter-individual variability and associated complexity constitutes a major experimental challenge but also presents an opportunity for microbiome research by enabling utilization of artificial intelligence and machine learning in decoding individualized patterns in the microbiome impacting on human health. As such, it will be intriguing to predict the ‘personalized’ host immune responses based on gut microbiome profiles, which will ultimately facilitate the development of personalized microbiome-targeted treatments for immunological diseases.

## Perspectives

A massive effort during the past decade in studying microbiome-immune interactions has led to better understanding of their molecular basis, while pointing to the importance of these interactions in impacting a variety of human immune-related diseases. Such insights are already spurring the development of microbiome-targeted therapeutic strategies in immune-mediated diseases. For example,  in an aim to restore a healthy microbiome configuration in patients suffering from dysbiosis linked to immune-mediated disease,  fecal microbiome transplantation (FMT), which has so far been widely used in *Clostridium difficile* infections, is considered also as potential treatment in this clinical context. However, there is still no general consensus on which features constitute a ‘healthy’ microbiome. The efficacy of FMT in diseases such as IBD, is therefore still under evaluation and many challenges remain to be overcome, including optimization of fecal processing and patient safety. Given that the prophylactic and therapeutic efficacy of traditional individual probiotics in promoting human health is limited, the use of ‘next-generation probiotics’, or rationally defined microbial consortia, potentially may provide a promising alternative.^263^ In addition to modalities aimed at replacing an entire microbiome, new techniques are aimed at editing the microbiome in a more precise way.^264^ For example, selective and precise depletion of certain pathobionts by bacteriophage therapy is being actively pursued.^265^ Diet-based alteration in nutrient availability may   constitute another feasible microbiome-modulating approach, given the strong influence of diet on gut microbiome composition and function. It may be intriguing to determine the efficacy of personalized diets, selective diets or manipulation of dietary timing in treating immunological disease, and to investigate how these diets influence host immune responses.^266^ Additionally, the large wealth of microbiome-derived metabolites found in high concentration throughout the gut and in the systemic circulation may offer an opportunity to modulate these potentially bioactive molecules (also called 'postbiotics'). Their supplementation or signaling blockade in defined immune contexts may offer new avenues of microbiome-directed treatments.^267^ Chemical genetic screening of gut microbiome metabolites^268^ might facilitate identification of bioactive metabolites that are important for host physiology or are implicated in immune-mediated diseases. Collectively, development of these microbiome-based therapies necessitates an enhanced understanding of the complex and intricate interactions between the microbiome and immunity. A successful translation of microbiome-based treatments  into clinical practice requires standardized, stringent and unbiased preclinical and clinical intervention studies.

## References

[1] Sender R, Fuchs S, Milo R (2016). Are we really vastly outnumbered? revisiting the ratio of bacterial to host cells in humans. Cell.

[2] Integrative HMP (iHMP) Research Network Consortium (2019). The integrative human microbiome project. Nature.

[3] Hacquard S (2015). Microbiota and host nutrition across plant and animal kingdoms. Cell Host Microbe.

[4] Lynch JB, Hsiao EY (2019). Microbiomes as sources of emergent host phenotypes. Science.

[5] Dethlefsen L, McFall-Ngai M, Relman DA (2007). An ecological and evolutionary perspective on human-microbe mutualism and disease. Nature.

[6] Macpherson AJ, Geuking MB, McCoy KD (2005). Immune responses that adapt the intestinal mucosa to commensal intestinal bacteria. Immunology.

[7] Chu H, Mazmanian SK (2013). Innate immune recognition of the microbiota promotes host-microbial symbiosis. Nat. Immunol..

[8] Zhang M (2017). Interactions between intestinal microbiota and host immune response in inflammatory bowel disease. Front. Immunol..

[9] Valitutti F, Cucchiara S, Fasano A (2019). Celiac disease and the microbiome. Nutrients.

[10] Maeda Y, Takeda K (2019). Host-microbiota interactions in rheumatoid arthritis. Exp. Mol. Med..

[11] Belizario JE, Faintuch J, Garay-Malpartida M (2018). Gut microbiome dysbiosis and immunometabolism: New frontiers for treatment of metabolic diseases. Mediators Inflamm..

[12] Main BS, Minter MR (2017). Microbial immuno-communication in neurodegenerative diseases. Front. Neurosci..

[13] Gopalakrishnan V, Helmink BA, Spencer CN, Reuben A, Wargo JA (2018). The influence of the gut microbiome on cancer, immunity, and cancer immunotherapy. Cancer Cell.

[14] Maynard CL, Elson CO, Hatton RD, Weaver CT (2012). Reciprocal interactions of the intestinal microbiota and immune system. Nature.

[15] Belkaid Y, Harrison OJ (2017). Homeostatic immunity and the microbiota. Immunity.

[16] Belkaid Y, Hand TW (2014). Role of the microbiota in immunity and inflammation. Cell.

[17] Gensollen T, Iyer SS, Kasper DL, Blumberg RS (2016). How colonization by microbiota in early life shapes the immune system. Science.

[18] Backhed F (2015). Dynamics and stabilization of the human gut microbiome during the first year of life. Cell Host Microbe.

[19] Koenig JE (2011). Succession of microbial consortia in the developing infant gut microbiome. Proc. Natl. Acad. Sci. USA.

[20] Yatsunenko T (2012). Human gut microbiome viewed across age and geography. Nature.

[21] Russell SL (2012). Early life antibiotic-driven changes in microbiota enhance susceptibility to allergic asthma. EMBO Rep..

[22] Zhang X, Zhivaki D, Lo-Man R (2017). Unique aspects of the perinatal immune system. Nat. Rev. Immunol..

[23] Bhutta ZA, Black RE (2013). Global maternal, newborn, and child health - So near and yet so far. N. Engl. J. Med..

[24] Neu J, Walker WA (2011). Necrotizing enterocolitis. N. Engl. J. Med..

[25] Wang J (2018). Dysbiosis of maternal and neonatal microbiota associated with gestational diabetes mellitus. Gut.

[26] Gomez de Aguero M (2016). The maternal microbiota drives early postnatal innate immune development. Science.

[27] Dominguez-Bello MG (2010). Delivery mode shapes the acquisition and structure of the initial microbiota across multiple body habitats in newborns. Proc. Natl. Acad. Sci. USA.

[28] Caballero-Flores G (2019). Maternal immunization confers protection to the offspring against an attaching and effacing pathogen through delivery of IgG in breast milk. Cell Host Microbe.

[29] Zheng W (2020). Microbiota-targeted maternal antibodies protect neonates from enteric infection. Nature.

[30] Bauer H, Horowitz RE, Levenson SM, Popper H (1963). The response of the lymphatic tissue to the microbial flora. Studies on germfree mice. Am. J. Pathol..

[31] Umesaki Y, Setoyama H, Matsumoto S, Okada Y (1993). Expansion of alpha beta T-cell receptor-bearing intestinal intraepithelial lymphocytes after microbial colonization in germ-free mice and its independence from thymus. Immunology.

[32] Hapfelmeier S (2010). Reversible microbial colonization of germ-free mice reveals the dynamics of IgA immune responses. Science.

[33] Ivanov II (2008). Specific microbiota direct the differentiation of IL-17-producing T-helper cells in the mucosa of the small intestine. Cell Host Microbe.

[34] Ivanov II (2009). Induction of intestinal Th17 cells by segmented filamentous bacteria. Cell.

[35] Tan TG (2016). Identifying species of symbiont bacteria from the human gut that, alone, can induce intestinal Th17 cells in mice. Proc. Natl. Acad. Sci. USA.

[36] Atarashi K (2015). Th17 cell induction by adhesion of microbes to intestinal epithelial cells. Cell.

[37] Mazmanian SK, Liu CH, Tzianabos AO, Kasper DL (2005). An immunomodulatory molecule of symbiotic bacteria directs maturation of the host immune system. Cell.

[38] Wesemann DR (2013). Microbial colonization influences early B-lineage development in the gut lamina propria. Nature.

[39] Cahenzli J, Koller Y, Wyss M, Geuking MB, McCoy KD (2013). Intestinal microbial diversity during early-life colonization shapes long-term IgE levels. Cell Host Microbe.

[40] Fulde M (2018). Neonatal selection by Toll-like receptor 5 influences long-term gut microbiota composition. Nature.

[41] Mowat AM (2018). To respond or not to respond - a personal perspective of intestinal tolerance. Nat. Rev. Immunol..

[42] Konrad A, Cong Y, Duck W, Borlaza R, Elson CO (2006). Tight mucosal compartmentation of the murine immune response to antigens of the enteric microbiota. Gastroenterology.

[43] Belkaid Y, Naik S (2013). Compartmentalized and systemic control of tissue immunity by commensals. Nat. Immunol..

[44] Shan M (2013). Mucus enhances gut homeostasis and oral tolerance by delivering immunoregulatory signals. Science.

[45] Bansal T, Alaniz RC, Wood TK, Jayaraman A (2010). The bacterial signal indole increases epithelial-cell tight-junction resistance and attenuates indicators of inflammation. Proc. Natl. Acad. Sci. USA.

[46] Peterson DA, McNulty NP, Guruge JL, Gordon JI (2007). IgA response to symbiotic bacteria as a mediator of gut homeostasis. Cell Host Microbe.

[47] Macpherson AJ, Uhr T (2004). Induction of protective IgA by intestinal dendritic cells carrying commensal bacteria. Science.

[48] Bevins CL, Salzman NH (2011). Paneth cells, antimicrobial peptides and maintenance of intestinal homeostasis. Nat. Rev. Microbiol..

[49] Ehmann D (2019). Paneth cell α-defensins HD-5 and HD-6 display differential degradation into active antimicrobial fragments. Proc. Natl. Acad. Sci. USA.

[50] Ahuja M (2017). Orai1-mediated antimicrobial secretion from pancreatic acini shapes the gut microbiome and regulates gut innate immunity. Cell Metab..

[51] Rakoff-Nahoum S, Paglino J, Eslami-Varzaneh F, Edberg S, Medzhitov R (2004). Recognition of commensal microflora by toll-like receptors is required for intestinal homeostasis. Cell.

[52] Price AE (2018). A map of Toll-like receptor expression in the intestinal epithelium reveals distinct spatial, cell type-specific, and temporal patterns. Immunity.

[53] Carvalho FA (2012). Transient inability to manage proteobacteria promotes chronic gut inflammation in TLR5-deficient mice. Cell Host Microbe.

[54] Vijay-Kumar M (2010). Metabolic syndrome and altered gut microbiota in mice lacking Toll-like receptor 5. Science.

[55] Ubeda C (2012). Familial transmission rather than defective innate immunity shapes the distinct intestinal microbiota of TLR-deficient mice. J. Exp. Med..

[56] Wen L (2008). Innate immunity and intestinal microbiota in the development of type 1 diabetes. Nature.

[57] Mazmanian SK, Round JL, Kasper DL (2008). A microbial symbiosis factor prevents intestinal inflammatory disease. Nature.

[58] Lee YK (2018). The protective role of *Bacteroides fragilis* in a murine model of colitis-associated colorectal cancer. mSphere.

[59] Ramakrishna C (2019). Bacteroides fragilis polysaccharide A induces IL-10 secreting B and T cells that prevent viral encephalitis. Nat. Commun..

[60] Erturk-Hasdemir Deniz, Oh Sungwhan F., Okan Nihal A., Stefanetti Giuseppe, Gazzaniga Francesca S., Seeberger Peter H., Plevy Scott E., Kasper Dennis L. (2019). Symbionts exploit complex signaling to educate the immune system. Proceedings of the National Academy of Sciences.

[61] Brown GD (2006). Dectin-1: a signalling non-TLR pattern-recognition receptor. Nat. Rev. Immunol..

[62] Tang C (2015). Inhibition of Dectin-1 signaling ameliorates colitis by inducing *Lactobacillus*-mediated regulatory T cell expansion in the intestine. Cell Host Microbe.

[63] Bouskra D (2008). Lymphoid tissue genesis induced by commensals through NOD1 regulates intestinal homeostasis. Nature.

[64] Ramanan D, Tang MS, Bowcutt R, Loke P, Cadwell K (2014). Bacterial sensor Nod2 prevents inflammation of the small intestine by restricting the expansion of the commensal *Bacteroides vulgatus*. Immunity.

[65] Nigro G, Rossi R, Commere PH, Jay P, Sansonetti PJ (2014). The cytosolic bacterial peptidoglycan sensor Nod2 affords stem cell protection and links microbes to gut epithelial regeneration. Cell Host Microbe.

[66] Janeway CA, Medzhitov R (2002). Innate immune recognition. Annu. Rev. Immunol..

[67] Vaishnava S (2011). The antibacterial lectin RegIIIgamma promotes the spatial segregation of microbiota and host in the intestine. Science.

[68] Wang S (2015). MyD88 adaptor-dependent microbial sensing by regulatory T cells promotes mucosal tolerance and enforces commensalism. Immunity.

[69] Broz P, Dixit VM (2016). Inflammasomes: Mechanism of assembly, regulation and signalling. Nat. Rev. Immunol..

[70] Elinav E (2011). NLRP6 inflammasome regulates colonic microbial ecology and risk for colitis. Cell.

[71] Levy M (2015). Microbiota-modulated metabolites shape the intestinal microenvironment by regulating NLRP6 inflammasome signaling. Cell.

[72] Wlodarska M (2014). NLRP6 inflammasome orchestrates the colonic host-microbial interface by regulating goblet cell mucus secretion. Cell.

[73] Birchenough GM, Nystrom EE, Johansson ME, Hansson GC (2016). A sentinel goblet cell guards the colonic crypt by triggering Nlrp6-dependent Muc2 secretion. Science.

[74] Wang P (2015). Nlrp6 regulates intestinal antiviral innate immunity. Science.

[75] Gálvez EJC, Iljazovic A, Gronow A, Flavell R, Strowig T (2017). Shaping of intestinal microbiota in Nlrp6- and Rag2-deficient mice depends on community structure. Cell Rep..

[76] Castro-Dopico T (2019). Anti-commensal IgG drives intestinal inflammation and type 17 immunity in ulcerative colitis. Immunity.

[77] Seo SU (2015). Distinct commensals induce interleukin-1beta via NLRP3 inflammasome in inflammatory monocytes to promote intestinal inflammation in response to injury. Immunity.

[78] Wolf AJ, Underhill DM (2018). Peptidoglycan recognition by the innate immune system. Nat. Rev. Immunol..

[79] Ratsimandresy RA, Indramohan M, Dorfleutner A, Stehlik C (2017). The AIM2 inflammasome is a central regulator of intestinal homeostasis through the IL-18/IL-22/STAT3 pathway. Cell Mol. Immunol..

[80] Saha S (2010). Peptidoglycan recognition proteins protect mice from experimental colitis by promoting normal gut flora and preventing induction of interferon-gamma. Cell Host Microbe.

[81] Jing X (2014). Peptidoglycan recognition protein 3 and Nod2 synergistically protect mice from dextran sodium sulfate-induced colitis. J. Immunol..

[82] Franchi L (2006). Cytosolic flagellin requires Ipaf for activation of caspase-1 and interleukin 1beta in salmonella-infected macrophages. Nat. Immunol..

[83] Zhu H (2017). RNA virus receptor Rig-I monitors gut microbiota and inhibits colitis-associated colorectal cancer. J. Exp. Clin. Cancer Res..

[84] Hornung V, Hartmann R, Ablasser A, Hopfner KP (2014). OAS proteins and cGAS: unifying concepts in sensing and responding to cytosolic nucleic acids. Nat. Rev. Immunol..

[85] Chudnovskiy A (2016). Host-protozoan interactions protect from mucosal infections through activation of the inflammasome. Cell.

[86] Mosser DM, Edwards JP (2008). Exploring the full spectrum of macrophage activation. Nat. Rev. Immunol..

[87] Danne C (2017). A large polysaccharide produced by *Helicobacter hepaticus* induces an anti-inflammatory gene signature in macrophages. Cell Host Microbe.

[88] Schulthess J (2019). The short chain fatty acid butyrate imprints an antimicrobial program in macrophages. Immunity.

[89] Wu K (2020). Gut microbial metabolite trimethylamine N-oxide aggravates GVHD by inducing M1 macrophage polarization in mice. Blood.

[90] Constantinides MG, McDonald BD, Verhoef PA, Bendelac A (2014). A committed precursor to innate lymphoid cells. Nature.

[91] Gury-BenAri M (2016). The spectrum and regulatory landscape of intestinal innate lymphoid cells are shaped by the microbiome. Cell.

[92] Sonnenberg GF, Hepworth MR (2019). Functional interactions between innate lymphoid cells and adaptive immunity. Nat. Rev. Immunol..

[93] McDonald BD, Jabri B, Bendelac A (2018). Diverse developmental pathways of intestinal intraepithelial lymphocytes. Nat. Rev. Immunol..

[94] Chun E (2019). Metabolite-sensing receptor Ffar2 regulates colonic group 3 innate lymphoid cells and gut immunity. Immunity.

[95] Bostick JW (2019). Dichotomous regulation of group 3 innate lymphoid cells by nongastric Helicobacter species. Proc. Natl. Acad. Sci. USA.

[96] Guo X (2015). Innate lymphoid cells control early colonization resistance against intestinal pathogens through ID2-dependent regulation of the microbiota. Immunity.

[97] Rankin LC (2016). Complementarity and redundancy of IL-22-producing innate lymphoid cells. Nat. Immunol..

[98] Chua HH (2018). Intestinal dysbiosis featuring abundance of *Ruminococcus gnavus* associates with allergic diseases in infants. Gastroenterology.

[99] Sterlin D (2020). Human IgA binds a diverse array of commensal bacteria. J. Exp. Med..

[100] Sutherland DB, Suzuki K, Fagarasan S (2016). Fostering of advanced mutualism with gut microbiota by immunoglobulin A. Immunol. Rev..

[101] Kawamoto S (2014). Foxp3+ T cells regulate immunoglobulin A selection and facilitate diversification of bacterial species responsible for immune homeostasis. Immunity.

[102] Palm NW (2014). Immunoglobulin A coating identifies colitogenic bacteria in inflammatory bowel disease. Cell.

[103] Shulzhenko N (2011). Crosstalk between B lymphocytes, microbiota and the intestinal epithelium governs immunity versus metabolism in the gut. Nat. Med..

[104] Nagashima K (2017). Identification of subepithelial mesenchymal cells that induce IgA and diversify gut microbiota. Nat. Immunol..

[105] Arpaia N (2013). Metabolites produced by commensal bacteria promote peripheral regulatory T-cell generation. Nature.

[106] Atarashi K (2011). Induction of colonic regulatory T cells by indigenous clostridium species. Science.

[107] Smith PM (2013). The microbial metabolites, short-chain fatty acids, regulate colonic Treg cell homeostasis. Science.

[108] Hegazy AN (2017). Circulating and tissue-resident CD4+ T cells with reactivity to intestinal microbiota are abundant in healthy individuals and function is altered during inflammation. Gastroenterology.

[109] Miossec P, Kolls JK (2012). Targeting IL-17 and Th17 cells in chronic inflammation. Nat. Rev. Drug Discov..

[110] Omenetti S (2019). The intestine harbors functionally distinct homeostatic tissue-resident and inflammatory Th17 cells. Immunity.

[111] Naik S (2012). Compartmentalized control of skin immunity by resident commensals. Science.

[112] Dutzan N (2017). On-going mechanical damage from mastication drives homeostatic Th17 cell responses at the oral barrier. Immunity.

[113] Bedoui S, Heath WR, Mueller SN (2016). CD4(+) T-cell help amplifies innate signals for primary CD8(+) T-cell immunity. Immunol. Rev..

[114] Bachem A (2019). Microbiota-derived short-chain fatty acids promote the memory potential of antigen-activated CD8(+) T cells. Immunity.

[115] Song X (2020). Microbial bile acid metabolites modulate gut RORgamma(+) regulatory T cell homeostasis. Nature.

[116] Crotty S (2014). T follicular helper cell differentiation, function, and roles in disease. Immunity.

[117] Kawamoto S (2012). The inhibitory receptor PD-1 regulates IgA selection and bacterial composition in the gut. Science.

[118] Proietti M (2014). ATP-gated ionotropic P2X7 receptor controls follicular T helper cell numbers in Peyer’s patches to promote host-microbiota mutualism. Immunity.

[119] Kubinak JL (2015). MyD88 signaling in T cells directs IgA-mediated control of the microbiota to promote health. Cell Host Microbe.

[120] Teng F (2016). Gut microbiota drive autoimmune arthritis by promoting differentiation and migration of Peyer’s patch T follicular helper cells. Immunity.

[121] Rescigno M, Rotta G, Valzasina B, Ricciardi-Castagnoli P (2001). Dendritic cells shuttle microbes across gut epithelial monolayers. Immunobiology.

[122] Martinez-Lopez M (2019). Microbiota sensing by Mincle-Syk axis in dendritic cells regulates interleukin-17 and -22 production and promotes intestinal barrier integrity. Immunity.

[123] Jie Z (2018). NIK signaling axis regulates dendritic cell function in intestinal immunity and homeostasis. Nat. Immunol..

[124] Wingender G (2012). Neutrophilic granulocytes modulate invariant NKT cell function in mice and humans. J. Immunol..

[125] An D (2014). Sphingolipids from a symbiotic microbe regulate homeostasis of host intestinal natural killer T cells. Cell.

[126] Rothschild D (2018). Environment dominates over host genetics in shaping human gut microbiota. Nature.

[127] Vojdani A (2014). A potential link between environmental triggers and autoimmunity. Autoimmune Dis..

[128] Yamamoto-Hanada K, Yang L, Narita M, Saito H, Ohya Y (2017). Influence of antibiotic use in early childhood on asthma and allergic diseases at age 5. Ann. Allergy Asthma Immunol..

[129] Becattini S, Taur Y, Pamer EG (2016). Antibiotic-induced changes in the intestinal microbiota and disease. Trends Mol. Med..

[130] Sato H (2016). Antibiotics suppress activation of intestinal mucosal mast cells and reduce dietary lipid absorption in Sprague-Dawley rats. Gastroenterology.

[131] Scott NA (2018). Antibiotics induce sustained dysregulation of intestinal T cell immunity by perturbing macrophage homeostasis. Sci. Transl. Med..

[132] Kim YG (2014). Gut dysbiosis promotes M2 macrophage polarization and allergic airway inflammation via fungi-induced PGE(2). Cell Host Microbe.

[133] Kim M (2018). Critical Role for the microbiota in CX3CR1(+) intestinal mononuclear phagocyte regulation of intestinal T cell responses. Immunity.

[134] Ohnmacht C (2015). MUCOSAL IMMUNOLOGY. The microbiota regulates type 2 immunity through RORgammat(+) T cells. Science.

[135] Hagan T (2019). Antibiotics-driven gut microbiome perturbation alters immunity to vaccines in humans. Cell.

[136] Christ A, Lauterbach M, Latz E (2019). Western diet and the immune system: An inflammatory connection. Immunity.

[137] Devkota S (2012). Dietary-fat-induced taurocholic acid promotes pathobiont expansion and colitis in Il10−/− mice. Nature.

[138] Cheng L (2016). High fat diet exacerbates dextran sulfate sodium induced colitis through disturbing mucosal dendritic cell homeostasis. Int. Immunopharmacol..

[139] Haghikia A (2015). Dietary fatty acids directly impact central nervous system autoimmunity via the small intestine. Immunity.

[140] He B (2017). Resetting microbiota by Lactobacillus reuteri inhibits T reg deficiency-induced autoimmunity via adenosine A2A receptors. J. Exp. Med..

[141] Rodriguez-Palacios A (2018). The artificial sweetener splenda promotes gut Proteobacteria, dysbiosis, and myeloperoxidase reactivity in Crohn’s disease-like ileitis. Inflamm. Bowel Dis..

[142] Viennois E, Merlin D, Gewirtz AT, Chassaing B (2017). Dietary emulsifier-induced low-grade inflammation promotes colon carcinogenesis. Cancer Res..

[143] Martinez I (2013). Gut microbiome composition is linked to whole grain-induced immunological improvements. ISME J..

[144] Cignarella F (2018). Intermittent fasting confers protection in CNS autoimmunity by altering the gut microbiota. Cell Metab..

[145] Rangan P (2019). Fasting-mimicking diet modulates microbiota and promotes intestinal regeneration to reduce inflammatory bowel disease pathology. Cell Rep..

[146] Bishehsari F (2020). Abnormal eating patterns cause circadian disruption and promote alcohol-associated colon carcinogenesis. Cell Mol. Gastroenterol. Hepatol..

[147] Rosshart SP (2019). Laboratory mice born to wild mice have natural microbiota and model human immune responses. Science.

[148] Kaplan GG (2015). The global burden of IBD: from 2015 to 2025. Nat. Rev. Gastroenterol. Hepatol..

[149] Kostic AD, Xavier RJ, Gevers D (2014). The microbiome in inflammatory bowel disease: current status and the future ahead. Gastroenterology.

[150] Gevers D (2014). The treatment-naive microbiome in new-onset Crohn’s disease. Cell Host Microbe.

[151] Franzosa EA (2019). Gut microbiome structure and metabolic activity in inflammatory bowel disease. Nat. Microbiol..

[152] Lloyd-Price J (2019). Multi-omics of the gut microbial ecosystem in inflammatory bowel diseases. Nature.

[153] de Souza HS, Fiocchi C (2016). Immunopathogenesis of IBD: current state of the art. Nat. Rev. Gastroenterol. Hepatol..

[154] Martini E, Krug SM, Siegmund B, Neurath MF, Becker C (2017). Mend your fences: The epithelial barrier and its relationship with mucosal immunity in inflammatory bowel disease. Cell Mol. Gastroenterol. Hepatol..

[155] Van der Sluis M (2006). Muc2-deficient mice spontaneously develop colitis, indicating that MUC2 is critical for colonic protection. Gastroenterology.

[156] Liso M (2020). A specific mutation in Muc2 determines early dysbiosis in colitis-prone Winnie mice. Inflamm. Bowel Dis..

[157] Ogura Y (2001). A frameshift mutation in NOD2 associated with susceptibility to Crohn’s disease. Nature.

[158] Hugot JP (2001). Association of NOD2 leucine-rich repeat variants with susceptibility to Crohn’s disease. Nature.

[159] Petnicki-Ocwieja T (2009). Nod2 is required for the regulation of commensal microbiota in the intestine. Proc. Natl. Acad. Sci. USA.

[160] Cadwell K (2008). A key role for autophagy and the autophagy gene Atg16l1 in mouse and human intestinal Paneth cells. Nature.

[161] Aden K (2018). ATG16L1 orchestrates interleukin-22 signaling in the intestinal epithelium via cGAS-STING. J. Exp. Med..

[162] Seregin SS (2017). NLRP6 protects Il10(−/−) mice from colitis by limiting colonization of Akkermansia muciniphila. Cell Rep..

[163] Schaubeck M (2016). Dysbiotic gut microbiota causes transmissible Crohn’s disease-like ileitis independent of failure in antimicrobial defence. Gut.

[164] Britton GJ (2019). Microbiotas from humans with inflammatory bowel disease alter the balance of gut Th17 and RORgammat(+) regulatory T cells and exacerbate colitis in mice. Immunity.

[165] Caruso R (2019). A specific gene-microbe interaction drives the development of Crohn’s disease-like colitis in mice. Sci. Immunol..

[166] Atarashi K (2017). Ectopic colonization of oral bacteria in the intestine drives TH1 cell induction and inflammation. Science.

[167] Torres J (2020). Infants born to mothers with IBD present with altered gut microbiome that transfers abnormalities of the adaptive immune system to germ-free mice. Gut.

[168] Scher JU (2013). Expansion of intestinal Prevotella copri correlates with enhanced susceptibility to arthritis. Elife.

[169] Maeda Y (2016). Dysbiosis contributes to arthritis development via activation of autoreactive T cells in the intestine. Arthritis Rheumatol..

[170] Alpizar-Rodriguez D (2019). Prevotella copri in individuals at risk for rheumatoid arthritis. Ann. Rheum. Dis..

[171] Chen J (2016). An expansion of rare lineage intestinal microbes characterizes rheumatoid arthritis. Genome Med..

[172] Zhang X (2015). The oral and gut microbiomes are perturbed in rheumatoid arthritis and partly normalized after treatment. Nat. Med..

[173] Wang Q, Xu R (2019). Data-driven multiple-level analysis of gut-microbiome-immune-joint interactions in rheumatoid arthritis. BMC Genom..

[174] Abdollahi-Roodsaz S (2008). Stimulation of TLR2 and TLR4 differentially skews the balance of T cells in a mouse model of arthritis. J. Clin. Invest..

[175] Rogier R (2017). Aberrant intestinal microbiota due to IL-1 receptor antagonist deficiency promotes IL-17- and TLR4-dependent arthritis. Microbiome.

[176] Wu HJ (2010). Gut-residing segmented filamentous bacteria drive autoimmune arthritis via T helper 17 cells. Immunity.

[177] de Aquino SG (2014). Periodontal pathogens directly promote autoimmune experimental arthritis by inducing a TLR2- and IL-1-driven Th17 response. J. Immunol..

[178] Hotamisligil GS (2017). Inflammation, metaflammation and immunometabolic disorders. Nature.

[179] Tilg H, Zmora N, Adolph TE, Elinav E (2020). The intestinal microbiota fuelling metabolic inflammation. Nat. Rev. Immunol..

[180] Kolodziejczyk AA, Zheng D, Shibolet O, Elinav E (2019). The role of the microbiome in NAFLD and NASH. EMBO Mol. Med..

[181] Henao-Mejia J (2012). Inflammasome-mediated dysbiosis regulates progression of NAFLD and obesity. Nature.

[182] Bodogai M (2018). Commensal bacteria contribute to insulin resistance in aging by activating innate B1a cells. Sci. Transl. Med..

[183] Virtue AT (2019). The gut microbiota regulates white adipose tissue inflammation and obesity via a family of microRNAs. Sci. Transl. Med..

[184] Truax AD (2018). The inhibitory innate immune sensor NLRP12 maintains a threshold against obesity by regulating gut microbiota homeostasis. Cell Host Microbe.

[185] Koeth RA (2019). l-Carnitine in omnivorous diets induces an atherogenic gut microbial pathway in humans. J. Clin. Invest..

[186] Wang Z (2011). Gut flora metabolism of phosphatidylcholine promotes cardiovascular disease. Nature.

[187] Gur C (2015). Binding of the Fap2 protein of Fusobacterium nucleatum to human inhibitory receptor TIGIT protects tumors from immune cell attack. Immunity.

[188] Mima K (2015). Fusobacterium nucleatum and T cells in colorectal carcinoma. JAMA Oncol..

[189] Ma C (2018). Gut microbiome-mediated bile acid metabolism regulates liver cancer via NKT cells. Science.

[190] Matson V (2018). The commensal microbiome is associated with anti-PD-1 efficacy in metastatic melanoma patients. Science.

[191] Gopalakrishnan V (2018). Gut microbiome modulates response to anti-PD-1 immunotherapy in melanoma patients. Science.

[192] Sivan A (2015). Commensal Bifidobacterium promotes antitumor immunity and facilitates anti-PD-L1 efficacy. Science.

[193] Routy B (2018). Gut microbiome influences efficacy of PD-1-based immunotherapy against epithelial tumors. Science.

[194] Vetizou M (2015). Anticancer immunotherapy by CTLA-4 blockade relies on the gut microbiota. Science.

[195] Viaud S (2013). The intestinal microbiota modulates the anticancer immune effects of cyclophosphamide. Science.

[196] Zitvogel L, Ayyoub M, Routy B, Kroemer G (2016). Microbiome and anticancer immunosurveillance. Cell.

[197] Pushalkar S (2018). The pancreatic cancer microbiome promotes oncogenesis by induction of innate and adaptive immune suppression. Cancer Discov..

[198] Geller LT (2017). Potential role of intratumor bacteria in mediating tumor resistance to the chemotherapeutic drug gemcitabine. Science.

[199] Riquelme E (2019). Tumor microbiome diversity and composition influence pancreatic cancer outcomes. Cell.

[200] Grice EA (2009). Topographical and temporal diversity of the human skin microbiome. Science.

[201] Oh J, Byrd AL, Park M, Kong HH, Segre JA (2016). Temporal stability of the human skin microbiome. Cell.

[202] Oh J (2014). Biogeography and individuality shape function in the human skin metagenome. Nature.

[203] Chehoud C (2013). Complement modulates the cutaneous microbiome and inflammatory milieu. Proc. Natl. Acad. Sci. USA.

[204] Brandwein M, Bentwich Z, Steinberg D (2017). Endogenous antimicrobial peptide expression in response to bacterial epidermal colonization. Front. Immunol..

[205] Naik S (2015). Commensal-dendritic-cell interaction specifies a unique protective skin immune signature. Nature.

[206] Linehan JL (2018). Non-classical immunity controls microbiota impact on skin immunity and tissue repair. Cell.

[207] Lai Y (2009). Commensal bacteria regulate Toll-like receptor 3-dependent inflammation after skin injury. Nat. Med..

[208] Scharschmidt TC (2015). A wave of regulatory T cells into neonatal skin mediates tolerance to commensal microbes. Immunity.

[209] Scharschmidt TC (2017). Commensal microbes and hair follicle morphogenesis coordinately drive Treg migration into neonatal skin. Cell Host Microbe.

[210] Sanford JA (2016). Inhibition of HDAC8 and HDAC9 by microbial short-chain fatty acids breaks immune tolerance of the epidermis to TLR ligands. Sci. Immunol..

[211] Nakatsuji T (2017). Antimicrobials from human skin commensal bacteria protect against Staphylococcus aureus and are deficient in atopic dermatitis. Sci. Transl. Med..

[212] Kong HH (2012). Temporal shifts in the skin microbiome associated with disease flares and treatment in children with atopic dermatitis. Genome Res..

[213] Stehlikova Z (2019). Dysbiosis of skin microbiota in Psoriatic patients: co-occurrence of fungal and bacterial communities. Front. Microbiol..

[214] Belkaid Y, Segre JA (2014). Dialogue between skin microbiota and immunity. Science.

[215] Nakamura Y (2013). Staphylococcus delta-toxin induces allergic skin disease by activating mast cells. Nature.

[216] Uluckan O (2019). Cutaneous immune cell-microbiota interactions are controlled by epidermal JunB/AP-1. Cell Rep..

[217] Ichinohe T (2011). Microbiota regulates immune defense against respiratory tract influenza A virus infection. Proc. Natl. Acad. Sci. USA.

[218] Fagundes CT (2012). Transient TLR activation restores inflammatory response and ability to control pulmonary bacterial infection in germfree mice. J. Immunol..

[219] Trompette A (2018). Dietary fiber confers protection against flu by shaping Ly6c(−) patrolling monocyte hematopoiesis and CD8(+) T cell metabolism. Immunity.

[220] Steed AL (2017). The microbial metabolite desaminotyrosine protects from influenza through type I interferon. Science.

[221] Marsland BJ, Gollwitzer ES (2014). Host-microorganism interactions in lung diseases. Nat. Rev. Immunol..

[222] Gollwitzer ES (2014). Lung microbiota promotes tolerance to allergens in neonates via PD-L1. Nat. Med..

[223] Pattaroni C (2018). Early-life formation of the microbial and immunological environment of the human airways. Cell Host Microbe.

[224] Dickson RP, Martinez FJ, Huffnagle GB (2014). The role of the microbiome in exacerbations of chronic lung diseases. Lancet.

[225] Yadava K (2016). Microbiota promotes chronic pulmonary inflammation by enhancing IL-17A and autoantibodies. Am. J. Respir. Crit. Care Med..

[226] Segal LN (2016). Enrichment of the lung microbiome with oral taxa is associated with lung inflammation of a Th17 phenotype. Nat. Microbiol..

[227] Larsen JM (2015). Chronic obstructive pulmonary disease and asthma-associated *Proteobacteria*, but not commensal *Prevotella spp*., promote Toll-like receptor 2-independent lung inflammation and pathology. Immunology.

[228] Jin C (2019). Commensal microbiota promote lung cancer development via gammadelta T cells. Cell.

[229] Corbitt N (2013). Gut bacteria drive Kupffer cell expansion via MAMP-mediated ICAM-1 induction on sinusoidal endothelium and influence preservation-reperfusion injury after orthotopic liver transplantation. Am. J. Pathol..

[230] Chen J (2014). Natural killer T cells play a necessary role in modulating of immune-mediated liver injury by gut microbiota. Sci. Rep..

[231] Liang S, Webb T, Li Z (2014). Probiotic antigens stimulate hepatic natural killer T cells. Immunology.

[232] Paik YH (2003). Toll-like receptor 4 mediates inflammatory signaling by bacterial lipopolysaccharide in human hepatic stellate cells. Hepatology.

[233] Nakamoto N (2019). Gut pathobionts underlie intestinal barrier dysfunction and liver T helper 17 cell immune response in primary sclerosing cholangitis. Nat. Microbiol..

[234] Liwinski T (2020). Alterations of the bile microbiome in primary sclerosing cholangitis. Gut.

[235] Yoshimoto S (2013). Obesity-induced gut microbial metabolite promotes liver cancer through senescence secretome. Nature.

[236] Loo TM (2017). Gut microbiota promotes obesity-associated liver cancer through PGE2-mediated suppression of antitumor immunity. Cancer Discov..

[237] Dapito DH (2012). Promotion of hepatocellular carcinoma by the intestinal microbiota and TLR4. Cancer Cell.

[238] Sharon G, Sampson TR, Geschwind DH, Mazmanian SK (2016). The central nervous system and the gut microbiome. Cell.

[239] Butovsky O, Weiner HL (2018). Microglial signatures and their role in health and disease. Nat. Rev. Neurosci..

[240] Erny D (2015). Host microbiota constantly control maturation and function of microglia in the CNS. Nat. Neurosci..

[241] Matcovitch-Natan O (2016). Microglia development follows a stepwise program to regulate brain homeostasis. Science.

[242] Thion MS (2018). Microbiome influences prenatal and adult microglia in a sex-specific manner. Cell.

[243] Abdel-Haq R, Schlachetzki JCM, Glass CK, Mazmanian SK (2019). Microbiome-microglia connections via the gut-brain axis. J. Exp. Med..

[244] Benakis C (2016). Commensal microbiota affects ischemic stroke outcome by regulating intestinal gammadelta T cells. Nat. Med..

[245] Ochoa-Reparaz J (2009). Role of gut commensal microflora in the development of experimental autoimmune encephalomyelitis. J. Immunol..

[246] Kim S (2017). Maternal gut bacteria promote neurodevelopmental abnormalities in mouse offspring. Nature.

[247] Reed MD (2020). IL-17a promotes sociability in mouse models of neurodevelopmental disorders. Nature.

[248] Yang D (2019). Dysregulated lung commensal bacteria drive interleukin-17B production to promote pulmonary fibrosis through their outer membrane vesicles. Immunity.

[249] O’Callaghan JL (2020). Re-assessing microbiomes in the low-biomass reproductive niche. BJOG.

[250] Minich JJ (2018). KatharoSeq enables high-throughput microbiome analysis from low-biomass samples. mSystems.

[251] Salter SJ (2014). Reagent and laboratory contamination can critically impact sequence-based microbiome analyses. BMC Biol..

[252] de Goffau MC (2019). Human placenta has no microbiome but can contain potential pathogens. Nature.

[253] Kuperman AA (2020). Deep microbial analysis of multiple placentas shows no evidence for a placental microbiome. BJOG.

[254] Karstens L (2019). Controlling for contaminants in low-biomass 16S rRNA gene sequencing experiments. mSystems.

[255] Burnham P (2020). Separating the signal from the noise in metagenomic cell-free DNA sequencing. Microbiome.

[256] Eisenhofer R (2019). Contamination in low microbial biomass microbiome studies: Issues and recommendations. Trends Microbiol..

[257] Limon JJ (2019). Malassezia is associated with Crohn’s disease and exacerbates colitis in mouse models. Cell Host Microbe.

[258] Sokol H (2017). Fungal microbiota dysbiosis in IBD. Gut.

[259] Norman JM (2015). Disease-specific alterations in the enteric virome in inflammatory bowel disease. Cell.

[260] Zuo T (2019). Gut mucosal virome alterations in ulcerative colitis. Gut.

[261] Liu TC, Stappenbeck TS (2016). Genetics and pathogenesis of inflammatory bowel disease. Annu. Rev. Pathol..

[262] Rosshart SP (2017). Wild mouse gut microbiota promotes host fitness and improves disease resistance. Cell.

[263] Tanoue T (2019). A defined commensal consortium elicits CD8 T cells and anti-cancer immunity. Nature.

[264] Zhu W (2018). Precision editing of the gut microbiota ameliorates colitis. Nature.

[265] Van Belleghem JD, Dabrowska K, Vaneechoutte M, Barr JJ, Bollyky PL (2018). Interactions between bacteriophage, bacteria, and the mammalian immune system. Viruses.

[266] Zeevi D (2015). Personalized nutrition by prediction of glycemic responses. Cell.

[267] Levy M, Thaiss CA, Elinav E (2016). Metabolites: messengers between the microbiota and the immune system. Genes Dev..

[268] Chen H (2019). A forward chemical genetic screen reveals gut microbiota metabolites that modulate host physiology. Cell.

